# Clinical validation of a DNA methylation biomarker associated with overall survival of relapsed ovarian cancer patients

**DOI:** 10.1002/ijc.70217

**Published:** 2025-11-01

**Authors:** Muhammad Habiburrahman, Nahal Masrour, Naina Patel, Anna M. Piskorz, Robert Brown, James D. Brenton, Iain A. McNeish, James M. Flanagan

**Affiliations:** ^1^ Division of Cancer, Department of Surgery and Cancer Imperial College London London UK; ^2^ Imperial Experimental Cancer Medicine Centre Imperial College London London UK; ^3^ Cancer Research UK Cambridge Institute University of Cambridge Cambridge UK; ^4^ Cambridge University Hospitals NHS Foundation Trust Cambridge UK; ^5^ Hammersmith Hospital Imperial College Healthcare NHS Foundation Trust London UK

**Keywords:** DNA methylation, epigenetic biomarker, ovarian cancer, platinum‐based chemotherapy, survival

## Abstract

Approximately 70% of ovarian cancer (OC) patients relapse after chemotherapy, underscoring the need to assess survival before second‐line treatment. We previously identified PLAT‐M8, an 8‐CpG blood‐based methylation signature linked to chemoresistance. This study validates its correlation with clinicopathological features and treatment profiles in additional cohorts. Extracted DNA from whole blood was provided from the BriTROC‐1 (*n* = 47) and OV04 cohorts (*n* = 57) upon the first relapse. Additional samples from Hammersmith Hospital (*n* = 100) were collected during first‐line chemotherapy (Cycles 3–4 and 6). Bisulphite pyrosequencing was used to quantify DNA methylation at the previously identified 8 CpG sites. The methylation data obtained were combined with previous data from ScoTROC‐1D and 1V (*n* = 141) and OCTIPS (*n* = 46). Cox regression was used to assess OS after relapse concerning clinicopathological characteristics. The DNA methylation Class (Class 1 vs. 2) was determined by consensus clustering. As for results, blood DNA methylation at relapse correlates with clinical outcomes, but it has no impact during first‐line treatment. Class 1 is linked to shorter survival (summary OS: HR 2.50, 1.64–3.79) and poorer prognosis on carboplatin monotherapy (OS: aHR 9.69, 95% CI: 2.38–39.47). It is associated with older (>75 years), advanced‐stage, platinum‐resistant patients, residual disease, and shorter PFS. In contrast, Class 2 is linked to platinum sensitivity, higher complete response rates (RECIST), and better prognosis but shows no correlation with CA‐125. These findings highlight PLAT‐M8's potential in guiding second‐line chemotherapy decisions. The PLAT‐M8 methylation biomarker is associated with survival in relapsed OC patients and may potentially predict their response to second‐line platinum treatment.

## Graphical abstract

What's new?

Most patients with ovarian cancer (OC) relapse after first‐line chemotherapy, and prognosis with second‐line treatment depends largely on the platinum‐free interval. However, reliable biomarkers to predict response to second‐line therapy remain lacking. Here, the authors examined associations between the DNA‐methylation biomarker PLAT‐M8 and survival in relapsed OC patients. Blood DNA methylation at relapse correlated with clinical outcome. In particular, hypomethylation was linked to shorter survival and features such as older age and advanced disease, while hypermethylation was associated with higher response rates and better prognosis. The findings support PLAT‐M8 as a relapse‐specific, non‐invasive marker for predicting clinical outcomes in relapsed OC.

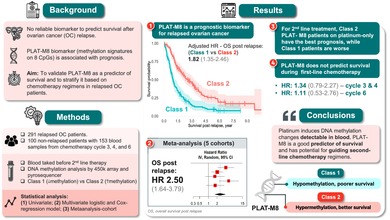

**Abbreviations**
aHR: adjusted hazard ratio
ARID5B: AT‐rich interaction domain 5B
ATG4A: autophagy related 4A cysteine peptidase
AUC: area under the curve
Bmi‐1 B: lymphoma Mo‐MLV insertion region 1 homolog
BriTROC‐1: British Translational Research Ovarian Cancer‐1
CA‐125: cancer antigen 125/carbohydrate antigen 125
CCC: clear‐cell carcinoma
CpG: cytosine‐phosphate‐guanine dinucleotide
CR: complete response
ctDNA: circulating tumour DNA
dsDNA: double‐stranded DNA
DUSP6: dual specificity phosphatase 6
ECOG: Eastern Cooperative Oncology Group Performance
FFPE: formalin‐fixed, paraffin‐embedded
FIGO: International Federation of Gynecology and Obstetrics
HGSOC: high‐grade serous ovarian carcinoma
HH: Hammersmith Hospital
HIST1H2BN: histone cluster 1 H2B family member N
ICHTB: Imperial College Healthcare Tissue Bank
KM: Kaplan–Meier
LGSOC: low‐grade serous ovarian carcinoma
MAD1L1: mitotic arrest deficient 1 like 1
MMR: mismatch repair
NPY: Neuropeptide Y
OC: ovarian cancer
OCTIPS: ovarian cancer therapy‐individualised treatment: project study
OS: overall survival
OV04: ovarian cancer studies which are part of the MRC trials series in the UK
PD: progressive disease
PFS: progression‐free survival
PLAT‐M8: an 8‐CpG blood‐based methylation signature linked to chemoresistance
PPP2R5E: protein phosphatase 2 regulatory subunit B'epsilon
PR: partial response
RD: residual disease
RECIST: response evaluation criteria in solid tumours
ROC: receiver operating characteristic curves
SAMD12: sterile alpha motif domain containing 12
SBNO2: strawberry notch homolog 2
ScoTROC‐1: Scottish Randomised Trial in Ovarian Cancer‐1
SD: stable disease
TFAP2E: transcription factor activating enhancer binding protein 2 epsilon
TFI/PFI: treatment/platinum‐free interval
ZNF385D: zinc finger protein 385D
ZPLD1: zona pellucida‐like domain containing 1

## 1 INTRODUCTION

Ovarian cancer (OC) has a high mortality rate in the United Kingdom and globally. One reason for this poor survival rate is that the majority of advanced OC cases relapse after optimal primary surgery and first‐line chemotherapy.^1^ Even among patients with homologous recombination deficiency (HRD+), who are generally more responsive to platinum‐based therapies, more than 50% still experience relapse, underscoring the limitations of current approaches.^2^ Platinum agents and poly(ADP‐ribose) polymerase inhibitors (PARPi) exploit overlapping DNA damage response pathways, and the introduction of PARPi has improved outcomes for HRD+ patients by delaying recurrence; however, resistance to PARPi eventually develops, and relapse remains common.^3^ Each relapse decreases overall survival (OS), worsening the prognosis.

The choice of second‐line treatment currently depends on the treatment/platinum‐free interval (TFI/PFI), which categorises recurrence as sensitive or resistant to chemotherapy based on the time since the last platinum treatment. This classification guides whether to use platinum monotherapy or in combination with other agents. A significant limitation of using the PFI to classify platinum sensitivity is the potential for biological misinterpretation. This time‐based metric primarily considers the duration since the last treatment and the time to disease progression, but it fails to account for the different histological characteristics. For instance, histological subtypes such as clear cell and mucinous carcinomas are known to be less sensitive to platinum‐based therapies, regardless of their progression timelines.^4^ Additionally, low‐grade serous ovarian carcinoma (LGSOC) exhibits inherent resistance to platinum due to its unique biological characteristics. The PFI approach also neglects the heterogeneous nature of tumour biology and variations in mismatch repair (MMR) capacity, both of which are crucial for understanding treatment responses and the development of chemoresistance. Misclassification could affect prognosis in relapsed patients receiving second‐line platinum. On the other hand, the current biomarker CA‐125 lacks specificity and consistency, underscoring the need for more reliable biomarkers in managing relapsed OC.^5^ Therefore, to improve treatment outcomes, there is a pressing need for more reliable biomarkers that reflect tumour behaviour through genomic and epigenomic analyses. These factors are essential for understanding the mechanisms of chemoresistance and ultimately guiding more personalised treatment strategies.

Evidence increasingly suggests that epigenetic alterations, such as DNA methylation, contribute to cancer development and can serve as reliable biomarkers for OC. Studies show that these biomarkers provide valuable insights into gene function and regulation in specific cell types. They account for external factors, such as therapy, hormones, nutrition, and lifestyle, which influence health and disease progression, acting as bioarchives.^6^ These markers are stable in various bodily fluids (plasma, serum, urine) and tissue samples (fresh, frozen, dried blood spots, FFPE). Research indicates that DNA integrity in plasma is maintained across different storage conditions, enabling reliable methylation analysis,^7^ while DNA from archived dried blood spots is suitable for genome‐wide profiling.^8^ Despite this, the role of epigenetics‐based biomarkers in cancer progression and chemotherapy resistance still requires further understanding. Epigenetic biomarkers are less invasive than tissue‐based ones and can identify relapse, assess prognosis, and evaluate treatment responses. Nevertheless, their clinical application remains limited as only a small number of studies have progressed these biomarkers to clinical validation. Our previous work identified a blood‐based DNA methylation signature on eight CpG sites, the PLAT‐M8 biomarker, which correlates with OS since diagnosis and post‐relapse.^8^ However, this initial study had a small sample size and did not explore its associations with clinicopathological characteristics.

Prognostic DNA methylation biomarkers in platinum‐based treatments require a full clinicopathological investigation, as these parameters may also affect methylation levels and treatment outcomes. Therefore, further validation is needed, and here, we aim to investigate PLAT‐M8's association with survival, second‐line treatment stratification, recurrence‐related parameters, and clinicopathological characteristics in relapsed OC patients across multiple UK cohorts. We also aim to determine whether this biomarker can be used earlier, during first‐line chemotherapy. The ultimate goal is to improve survival rates and reduce the harmful side effects in platinum‐nonresponsive patients.

## 2 MATERIALS AND METHODS

### 2.1 Study design, data source and samples

This validation study aimed to evaluate the prognostic value of PLAT‐M8 in relation to OS following OC relapse. Patients were included consecutively, and sample size was based on availability during the study period. Data on relapsed patients were obtained from subset datasets of prior cohorts, including blood DNA from two ScoTROC‐1 datasets: ScoTROC‐1V (*n* = 54) and ScoTROC‐1D (*n* = 87), originally part of ScoTROC‐1.^9^ Tissue DNA was sourced from OCTIPS (*n* = 46),^10^ previously used in Flanagan et al.,^8^ who first identified PLAT‐M8 in 2017. Two novel relapsed blood DNA datasets were added from BriTROC‐1 (*n* = 47)^11^ and OV04 (*n* = 57)^12^ as further independent validations. Additionally, blood DNA datasets of primary diagnostic (non‐relapsed) OC were prospectively collected from 153 new blood samples obtained from 100 patients at Hammersmith Hospital (HH) during first‐line chemotherapy cycles (Cycle 3: *n* = 28, Cycle 4: *n* = 75, Cycle 6: *n* = 49). The HH samples were collected to assess whether this biomarker could be used earlier, before relapse. While the OCTIPS cohort included tumour tissue DNA, the others used peripheral blood DNA; prior studies have shown that tumour and blood methylation patterns are similar.^8^ The procedures for blood and patient data collection are outlined in Figure S1, and additional details about data collection are provided in the Supporting Information Methods (Data source and samples).

### 2.2 Eligibility criteria and clinical variables

We analysed DNA from previously published cohorts and applied specific inclusion criteria to select cases, with further details provided in the Supporting Information Methods (Patient characteristics). These criteria included women diagnosed with epithelial OC at FIGO stage IC– IV, experiencing their first relapse, and receiving platinum. All blood samples (and tumour samples from OCTIPS which were from secondary‐debulking surgery) were available at the time of their first relapse before second‐line chemotherapy (except blood samples from HH). This study examined the clinicopathological profiles of patients with relapsed OC. Recurrence was evaluated using Response Evaluation Criteria in Solid Tumours (RECIST) and CA‐125 levels after first‐line therapy per Gynaecologic Cancer Intergroup guidelines. Patient ages at presentation and relapse were categorised per decade, with ‘younger patients’ defined as those aged 75 years old or younger, reflecting the peak rate of OC cases in the United Kingdom. Clinical staging was based on FIGO criteria, further divided into early stage (I–II) and advanced stage (III–IV). Histological subtypes and tumour grades followed WHO criteria for serous and non‐serous carcinoma. Chemotherapy regimens were categorised as ‘carboplatin monotherapy’ and ‘carboplatin with other therapies’ for first‐line treatment, while for second‐line treatment, they were classified as ‘carboplatin monotherapy’ and ‘other therapies with/without carboplatin’. PFI classification was simplified into platinum‐sensitive (≥6 months since last chemotherapy) or platinum‐resistant (<6 months since last chemotherapy). Surgical types and outcomes (e.g., residual disease/RD) were simplified, and categorised as either no RD or any RD, accounting for varying cutoffs across cohorts. Detailed explanations of the clinical variables in this study are provided in the Supporting Information Methods (Clinical variable descriptions).

Clinical endpoints included progression‐free survival (PFS), and OS after first relapse. Survival analyses (e.g., Cox regression) accounted for censoring due to loss to follow‐up. The biomarker status was classified into two classes based on clustering consensus of methylation scores of 8 CpG sites using the ‘*ConsensusClusterPlus*’ package in R based on cophenetic correlation coefficient assessment. Consensus clustering identified the optimal two clusters for PLAT‐M8: Class 1 (hypomethylated or not methylated) and Class 2 (hypermethylated). The biomarker status was then combined with second‐line chemotherapy into four categories: Class 2, carboplatin only; Class 2, other regimens ± carboplatin; Class 1, carboplatin only; and Class 1, other regimens ± carboplatin.

### 2.3 Methylation analysis

DNA samples from clinical whole blood were extracted using Qiagen DNA Blood Mini Kits (Qiagen QIAamp®, Manchester, UK) following the manufacturer's protocols. Quantification was conducted with a Qubit 2.0 fluorimeter (Life Technologies, Paisley, UK) using the dsDNA BR Assay. Bisulphite conversion of 500 ng of genomic DNA was performed using the EZ‐96 DNA‐methylation Gold kit (Zymo Research) according to the manufacturer's instructions. Methylation‐specific PCR and pyrosequencing were conducted as previously using the primers described in the Supporting Information Methods (Methylation analysis).

### 2.4 Statistical analysis

Subgroup analyses were performed by stratifying patients based on predefined clinical variables (e.g., relapse/non‐relapse, biomarker class, treatment group). The association between clinicopathological factors, mortality status, and biomarker status was assessed using *χ* ^2^, Fisher's exact, and non‐parametric tests in SPSS v29. Mann–Whitney and Kruskal‐Wallis tests evaluated age differences across groups. Life tables and the log‐rank test compared survival times and rates. Interaction effects were assessed using multivariable models that included interaction terms between biomarker status and relevant clinical covariates. Multivariate logistic regression identified factors linked to PLAT‐M8 Class 1, including age at relapse, FIGO stage, grade, histology, first‐line chemotherapy, residual tumour, and PFS which was evaluated using likelihood ratio tests. Class 1 and 2 distributions were compared using *χ* ^2^. Further analysis in R v4.3.1 and RStudio used Kaplan–Meier (KM) survival analysis in BriTROC‐1 and OV04, with log‐rank tests for group differences. In the ‘BriTROC‐1 + OV04 study’, OS was compared based on second‐line chemotherapy. Survival analysis was not conducted for ScoTROC‐1 (V and D) or OCTIPS, except for clinicopathological analyses, due to prior publications and missing second‐line chemotherapy data.^8^ Cox proportional hazards regression combined harmonised data from five validation subsets, with biomarker status and clinicopathological factors in a multivariate model. Univariate Cox regression HRs were assessed via meta‐analysis, with forest plots summarising OS effect size estimates and heterogeneity evaluation. Sensitivity analyses examined survival after relapse by progression time and histology. Prognostic value was assessed with time‐dependent survival receiver operating characteristic curves (ROCs). Statistical significance was set at *p* < .05. More detailed descriptions of the statistical analyses, including sensitivity analyses, are provided in the Supporting Information Methods (Statistical analysis).

## 3 RESULTS

### 3.1 Patient clinicopathological characteristics

We analysed DNA methylation from 391 patients and 444 blood samples (Table S1), revealing cohort‐specific variations in PLAT‐M8 classes, with Class 1 dominating ScoTROC‐1D and HH Cycle 6, Class 2 being more prevalent in OCTIPS, BriTROC‐1, OV04, and HH Cycles 3 and 4, and a balanced distribution observed in ScoTROC‐1V. Baseline characteristics are presented in Table S2. Among 291 relapsed cases (74.42%), age at diagnosis and relapse varied by cohort. The 2‐year relapse rate was 32%, highest in OCTIPS and lowest in ScoTROC‐1D, while HH non‐relapsed patients had a moderate 2‐year OS of 40%. Significant differences in PFS, OS, and follow‐up months were observed across five relapsed‐case cohorts. Non‐relapsed HH patients (*n* = 100, 25.58%) were slightly older at diagnosis, had longer OS, but shorter follow‐up from the initial diagnosis timepoint (63.3 months in non‐relapsed vs. 80.7 months in relapsed cases). Relapsed cases were mostly late‐stage (89.0% stage III–IV) with serous carcinoma (70.8%) as the dominant histology. Most tumours (92.6%) were poorly or undifferentiated (G3‐G4). For first‐line chemotherapy, 76.9% received platinum and taxane (carboplatin + paclitaxel), while other regimens were used in ScoTROC‐1D/1V, OCTIPS, and BriTROC‐1; whereas in second‐line treatment, 35.0% received platinum‐taxane regimens again. In the BriTROC‐1 and OV04 datasets, 48.1% of Class 2 and 33.7% of Class 1 patients received alternative regimens, with/without platinum, as second‐line therapy. In ScoTROC‐1D/1V, 51.1% had Eastern Cooperative Oncology Group Performance status (ECOG) 1, indicating limited physical activity but capable of light work. Most patients (91.2%) underwent interval debulking, 60.1% had RDs, 50% achieved complete response, and 77.7% showed CA‐125 decline. PLAT‐M8 was balanced in relapsed cases (6:5) but favoured Class 2 in non‐relapsed (7:5).

### 3.2 Association between clinicopathological features with mortality and biomarker status

As outcome events, we noted that in relapse cases, 208/291 (71.5%) patients had died, while in non‐relapse cases, 58/100 (58%) patients had died. Clinicopathological characteristics linked to mortality and biomarker status are summarised in Table 1 (full analyses in Tables S3 and S4). Among relapsed cases, mortality was significantly higher in advanced FIGO stages (*p* = .043), non‐serous carcinoma (*p* = .001), non‐carboplatin first‐line regimens (*p* = .047), platinum resistance (*p* = .039), RD presence (*p* = .033), shorter PFS (*p* = .010), and Class 1 PLAT‐M8 (*p* < .001). In non‐relapsed HH cases, no significant clinicopathological differences were found, but deceased patients had a higher rate of progressive disease per RECIST (*p* = .002). In relapsed cases, Class 1 PLAT‐M8 was more prevalent in patients over 75 (*p* = .015), advanced FIGO stages (*p* = .038), those with RD (*p* = .024), and those with shorter PFS (*p* < .001), also linking to platinum sensitivity and RECIST. Non‐relapsed HH cases showed no significant clinicopathological differences. Multivariate logistic regression (Table S5) identified FIGO stage, RD, and PFS as factors for Class 1, but after adjustment, only PFS remained significant (OR 6.33, *p* < .001).

**TABLE 1:** Patient characteristics and comparative analysis of clinicopathological features in five subsets of cohorts with relapsed ovarian cancer (*n* = 291) by mortality and biomarker status (significant results shown; full data in Supporting Information tables).

Clinicopathological features	Mortality status				Total		*p*‐value	Biomarker status				Total		*p*‐value
	Alive		Death					Class 1		Class 2				
	*n*	%	*n*	%	*n*	%		*n*	%	*n*	%	*n*	%	
Age at diagnosis														
21–30 years	0	0	4	100	4	1.4	.112^a^	3	75.0	1	25.0	4	1.4	.**044** ^a^
31–40 years	1	16.7	5	83.3	6	2.1		5	83.3	1	16.7	6	2.1	
41–50 years	13	29.5	31	70.5	44	15.1		26	59.1	18	40.9	44	15.1	
51–60 years	22	25.3	65	74.7	87	29.9		52	59.8	35	40.2	87	29.9	
60–70 years	28	26.9	76	73.1	104	35.7		50	48.1	54	51.9	104	35.7	
>70 years	19	41.3	27	58.7	46	15.8		23	50.0	23	50.0	46	15.8	
Age at diagnosis														
Younger (≤75 years)	74	27.6	194	72.4	268	92.1	.240^b^	152	56.7	116	43.3	268	92.1	.**015** ^b^
Elder (>75 years)	9	35.3	14	64.7	23	7.9		7	30.4	16	69.6	23	7.9	
FIGO stage														
I	9	64.3	5	35.7	14	4.8	.**023** ^b^	9	64.3	5	35.7	14	4.8	.129^b^
II	5	27.8	13	72.2	18	6.2		14	77.8	4	22.2	18	6.2	
III	55	27.5	145	72.5	200	68.7		108	54.0	92	46.0	200	68.7	
IV	14	23.7	45	76.3	59	20.3		28	47.5	31	52.5	59	20.3	
FIGO stage degree														
Early (I–II)	14	43.8	18	56.3	32	11.0	.**043** ^b^	23	71.9	9	28.1	32	11.0	.**038** ^b^
Advanced (III–IV)	69	26.6	190	73.4	259	89.0		136	52.5	123	47.5	259	89.0	
Histological subtypes														
Serous carcinoma	70	34.0	136	66.0	206	70.8	.**004** ^a^	119	57.8	93	42.2	206	70.8	.166^a^
Adenocarcinoma NOS	1	4.0	24	96.0	25	8.6		6	24.0	19	76.0	25	8.6	
Papillary adenocarcinoma	3	14.3	18	85.7	21	7.2		16	76.2	5	23.8	21	7.2	
Mucinous adenocarcinoma	1	25.0	3	75.0	4	1.4		1	25.0	3	75.0	4	1.4	
Endometrioid carcinoma	6	33.3	12	66.7	18	6.2		9	50.0	9	50.0	18	6.2	
Clear cell carcinoma	1	20.0	4	80.0	5	1.7		1	20.0	4	80.0	5	1.7	
Carcinosarcoma	0	0	0	0	0	0		0	0	0	0	0	0	
MCOA histological type	0	0	3	100	3	1.0		2	66.7	1	33.3	3	1.0	
Other ovarian malignancies	1	11.1	8	88.9	9	3.1		5	55.6	4	44.4	9	3.1	
Histological group														
Serous carcinoma	70	34.0	136	66.0	206	70.8	.**001** ^b^	119	57.8	87	42.2	206	70.8	.095^b^
Non‐serous carcinoma	13	15.3	72	84.7	85	29.2		40	47.1	45	52.9	85	29.2	
First‐line chemotherapy class														
Cp monotherapy	17	35.4	31	64.6	48	21.0	.**047** ^b^	31	64.6	17	35.4	48	21.0	.118^b^
Cp with other therapies	39	21.5	142	78.5	181	79.0		94	51.9	87	48.1	181	79.0	
Missing data					62							62		
Platinum sensitivity level														
Sensitive	17	42.5	23	57.5	40	85.1	.**039** ^c^	29	72.5	11	27.5	40	85.1	.**036** ^c^
Resistant	0	0	7	100	7	14.9		2	28.6	5	71.4	7	14.9	
Missing data					244							244		
Residual disease														
No residual disease	30	33.0	61	67.0	91	39.9	.**033** ^b^	57	62.6	34	37.4	91	39.9	.**024** ^b^
Any residual disease	28	20.4	109	79.6	137	60.1		65	47.4	72	52.6	137	60.1	
Missing data					63							63		
RECIST response														
Complete response	20	30.3	46	69.7	66	50.0	.149^b^	39	59.1	27	40.9	66	50.0	.**004** ^a^
Partial response	4	11.4	31	88.6	35	26.5		16	45.7	19	54.3	35	26.5	
Stable response	4	16.7	20	83.3	24	18.2		9	37.5	15	62.5	24	18.2	
Progressive disease	2	28.6	5	71.4	7	5.3		0	0	7	100	7	5.3	
Missing data					159							159		
PFS time for first relapse														
>327 days	68	32.9	139	67.1	207	71.1	.**010** ^b^	139	67.1	68	32.9	207	71.1	**<.001** ^b^
≤327 days	15	17.9	69	82.1	84	28.9		20	23.8	64	76.2	84	28.9	
Biomarker status														
Class 2	59	37.1	100	62.9	159	54.6	**<.001** ^b^							
Class 1	24	18.2	108	81.8	132	45.4								

*Note*: The percentage ‘%’ values in each group represent row percentages; meanwhile, the percentage ‘%’ values in the total represent column percentages. +/− Cp means with or without carboplatin since some patients did not receive carboplatin as their primary therapy, and ‘other’ means other regimens of chemotherapy besides carboplatin. Taxane (e.g., paclitaxel and docetaxel), TopII inhibitor (e.g., etoposide), ACs (e.g., liposomal doxorubicin and epirubicin), alkylating agents (e.g., cyclophosphamide), STKi (e.g., enzastaurin), EGFR‐TKi (e.g., erlotinib and sorafenib), anti‐VEGF (e.g., bevacizumab), TopI inhibitor (e.g., topotecan), antimetabolites (e.g., gemcitabine). The bold values indicate significant *p*‐values.

Abbreviations: ACs, anthracyclines; Cp, carboplatin; ECOG, Eastern Cooperative Oncology Group performance status; EGFR‐TKi, epidermal growth factor receptor tyrosine kinase inhibitor; FIGO, International Federation of Gynecology and Obstetrics; MCOA, mixed cell ovarian adenocarcinoma; NOS, non‐specific; Plat, platinum; RECIST, response evaluation criteria in solid tumours; STKi, serine–threonine kinase inhibitors; TopII, topoisomerase II; VEGF, vascular endothelial growth factor.

^a^ Mann–Whitney.

^b^ Chi‐square.

^c^ Fisher's exact test.

### 3.3 Independent validation of PLAT‐M8 in BriTROC‐1 and OV04

**FIGURE 1:**
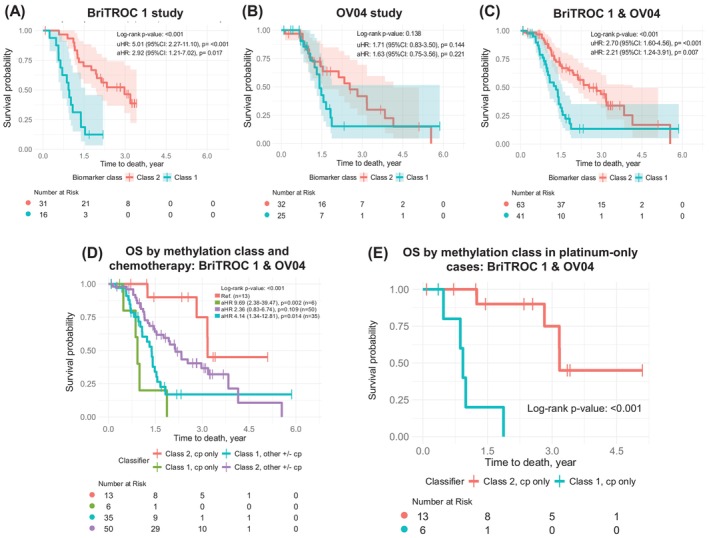
Clinical validation results of the PLAT‐M8 biomarker in two independent relapsed cohorts (BriTROC‐1 and OV04) as a prognostic marker for overall survival (OS) after first relapse and a potential predictor of second‐line platinum‐based chemotherapy response. (A) The Kaplan–Meier (KM) curve in BriTROC‐1 (*n* = 16, Class 1 in blue; *n* = 31, Class 2 in red) indicates a worse prognosis for Class 1 biomarkers compared to Class 2 (reference), with an adjusted hazard ratio (aHR) of 2.92 (95% CI: 1.21–7.02, *p* = .017), log‐rank *p* < .001. (B) OV04 (*n* = 25, Class 1 in blue; *n* = 32, Class 2 in red) KM curve shows no statistically significant difference in survival between Class 1 and Class 2 patients. Adjusted multivariable Cox regression for OS shows aHR of 1.63 (95% CI: 0.75–3.56, *p* = .221), log‐rank *p* = .138. (C) Adjusted multivariable Cox regression for OS in the combined dataset analysis of BriTROC‐1 and OV04 shows an aHR of 2.21 (95% CI: 1.24–3.91, *p* = .007), log‐rank *p* < .001. All adjustments were made for the covariates of age at relapse, cancer stage, histology, and progression‐free survival (PFS). (D) Using BriTROC‐1 and OV04 data, a KM curve for OS was created for each class, stratified by second‐line treatment. Patients who received carboplatin (Cp) alone as a single treatment in the Class 1 biomarker group (‘Class 1, Cp only’) or Class 2 biomarker group (‘Class 2, Cp only’) were examined in the survival curves. Other treatments, with or without Cp, were also analysed within Class 1 (‘Class 1, other +/− Cp’, *n* = 35, green) and Class 2 (‘Class 2, other +/− Cp’, *n* = 50, purple). These other regimens include paclitaxel (*n* = 56), liposomal doxorubicin (*n* = 21), gemcitabine (*n* = 9), cediranib (*n* = 3), epirubicin (*n* = 2), and bevacizumab (*n* = 2). This analysis used ‘Class 2, Cp only’ as the reference group. The OS post‐relapse analysis showed that ‘Class 2, Cp only’ had the best prognosis, whereas ‘Class 1, Cp only’ had the worst, with an aHR of 9.69 (95% CI: 2.38–39.47, *p* = .002). Overall log‐rank *p* < .001 indicated intermediate outcomes for ‘Class 1, other +/− Cp’ and ‘Class 2, other +/− Cp’ groups. (E) Comparing OS between Class 1 (*n* = 6) and Class 2 (*n* = 13) patients who received rechallenged platinum, the ‘Class 2, Cp only’ group showed a more favourable prognosis (log‐rank *p* < .001). All charts were truncated at 6 years for consistency. BriTROC‐1, British Translational Research Ovarian Cancer Collaborative 1; ECOG: Eastern Cooperative Oncology Group performance status; FIGO, International Federation of Gynecology and Obstetrics; HH, Hammersmith Hospital; OCTIPS, ovarian cancer therapy innovative models prolong survival; OS, overall survival after relapse; OV04, ovarian cancer clinical trial study 4th edition; PFS, progression‐free survival; PFI, platinum‐free interval; PFS, progression‐free survival; RECIST, response evaluation criteria in solid tumours; ScoTROC‐1, Scottish Randomised Trial in Ovarian Cancer (D, discovery and V, validation).

Our previous blood‐based DNA methylation study (using ScoTROC‐1 datasets) identified 8 CpG sites linked to survival, which were further validated in OCTIPS tissue biopsies.^8^ We extended validation to BriTROC‐1 and OV04 to assess its consistency. In BriTROC‐1, adjusted Cox regression showed worse OS for Class 1 vs. Class 2 (aHR 2.92, 1.21–7.02, *p* = .017; Figure 1A), while OV04 showed no OS difference (Figure 1B). Combined data (BriTROC‐1 + OV04) confirmed poorer OS for Class 1 (aHR 2.21, 1.24–3.91, *p* = .007; Figure 1C). Normalised methylation analysis (Figure S2) showed decreased methylation in ScoTROC‐1D‐450 K, aligning with other cohorts. Boxplot analysis (Figure S3) revealed significant hypermethylation at 4/8 CpG sites and hypomethylation at 1/8 in BriTROC‐1, with similar patterns in OV04. Stratifying OS by second‐line treatment (Table S6), Class 2 patients on carboplatin monotherapy had the best prognosis, while Class 1 had the worst (aHR 9.69, 2.38–39.47, *p* = .002; Figure 1D,E).

### 3.4 PLAT‐M8 lacks prognostic value during initial chemotherapy cycles

**FIGURE 2:**
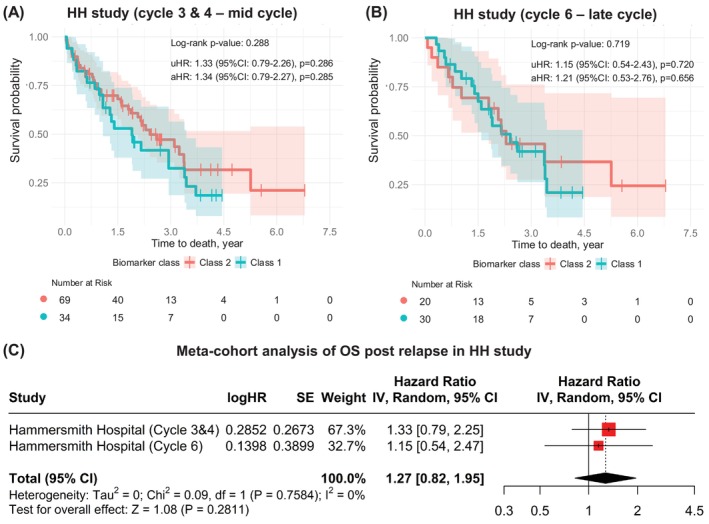
PLAT‐M8 validation in the Hammersmith Hospital (HH) cohort of primary ovarian cancer patients during first‐line chemotherapy (prior to relapse). (A) Focusing on cycle‐specific data, in HH Cycles 3 and 4 (*n* = 16, Class 1 in blue; *n* = 31, Class 2 in red), OS analysis showed no significant difference (aHR 1.34, 95% CI: 0.79–2.27, *p* = .285; KM log‐rank *p* = .288). (B) In HH Cycle 6 (*n* = 25, Class 1 in blue; *n* = 32, Class 2 in red), OS analysis remained insignificant (aHR 1.21, 95% CI: 0.53–2.76, *p* = .656; KM log‐rank *p* = .719), though Class 1 showed a tendency for worse survival. Hazard ratios were adjusted for age, cancer stage, histology, and progression‐free survival (PFS). (C) In non‐relapse cases, the meta‐cohort analysis remained insignificant, with OS summary HR 1.27 (95% CI: 0.82–1.95, *p* = .28, *I* ^2^ = 0%, *p* = .76).

We investigated whether PLAT‐M8, effective at first relapse, also predicts outcomes when measured earlier at primary diagnosis during first‐line chemotherapy. Data from 100 OC patients (HH cohort) showed no significant clinicopathological differences or prognostic value for OS (Tables S3 and S4, and Figure S4). Multivariate Cox regression (Table S7) confirmed no OS association. Looking into different cycles in 153 samples of the HH cohort, PLAT‐M8 showed no prognostic value for OS in Cycles 3 and 4 or Cycle 6 (aHR 1.34, *p* = .285; aHR 1.21, *p* = .656; Figure 2A,B). Meta‐cohort analysis (Figure 2C) confirmed no association between Class 1 and poorer prognosis. While methylation differences at specific CpG sites in the HH cohort (Figure S3) were noted, they were inconsistent with relapsed cases.

### 3.5 Summary of existing evidence on PLAT‐M8 detection at relapse as a prognostic factor

**FIGURE 3:**
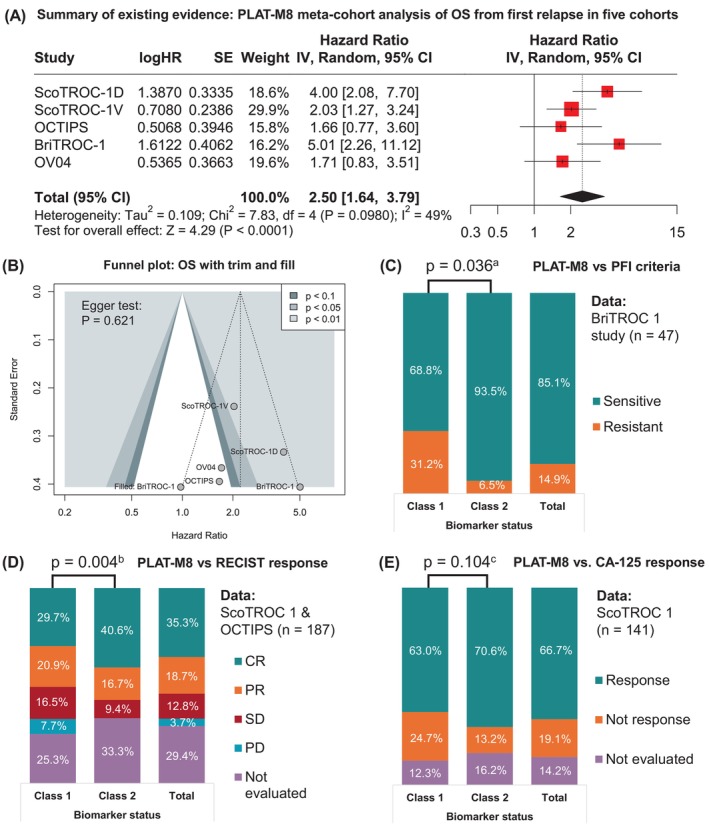
Summary of existing evidence of PLAT‐M8 testing across different relapsed case cohorts and its association with relapse‐related clinical parameters. (A) The OS analysis in the meta‐cohort accounting for heterogeneity showed that Class 1 of PLAT‐M8 had a poorer prognosis (HR: 2.50, 95% CI: 1.64–3.79, *p* < .0001) with no significant heterogeneity (*I* ^2^ = 49%, *p* = .0098). (B) A contoured funnel plot for OS analysis in five relapsed cohorts showed no significant publication bias (Egger's test) and relative symmetry, with most studies in the highly significant area (*p* < .01). Trim‐and‐fill analysis suggested adding one study for symmetry (black triangle). (C) PLAT‐M8 was validated with recurrence‐related parameters. In BriTROC‐1 (PFI data: Class 1, *n* = 16; Class 2, *n* = 31), Class 2 was linked to platinum sensitivity (^a^Fisher's exact test, *p* = .036). (D) In ScoTROC‐1 and OCTIPS (RECIST response: Class 1, *n* = 68; Class 2, *n* = 64), Class 2 had more complete responders (^b^Mann–Whitney test, *p* = .004). (E) In ScoTROC‐1 (CA‐125 response: Class 1, *n* = 64; Class 2, *n* = 57), Class 2 tended to have a higher proportion of responsive patients (^c^ *χ* ^2^ test, *p* = .104).

To summarise the current testing of this biomarker, we combined all relapsed‐case cohorts (blood and tumour tissue samples) into a meta‐cohort analysis using univariate Cox regression with a random‐effect model. This accounted for heterogeneity in OS (Figure 3A) and yielded a summary HR of 2.50 (1.64–3.79, *p* < .0001), with no significant heterogeneity detected and no publication bias per the Egger test (Figure 3B). Despite few studies, relative symmetry was observed. Trim and fill analysis indicated potential missing OS studies, but without significant impact on results. The meta‐cohort's summary HR is higher than the combined KM survival analysis of five harmonised datasets (aHR 1.82, 1.35–2.46, *p* < .001) (Table S8 and Figure S5). Multivariate Cox regression also identified advanced stage (aHR 1.87, *p* = .014), non‐serous histology (aHR 1.82, *p* < .001), and PFS ≤327 days (aHR 1.87, *p* < .001) as prognostic factors.

In a more detailed subanalysis by time points and clinicopathological characteristics, OS differences between PLAT‐M8 classes were most pronounced in the first 2 years of relapse, but follow‐up time between the two classes did not differ (Table S9). Median PFS and OS varied by clinicopathological factors, with Class 1 showing poorer survival than Class 2 (Table S10). Blood DNA methylation at relapse correlated with outcomes, with Class 2 having an 18.8‐month longer median OS—over 2.6 times that of Class 1. Advanced stage (III–IV) was linked to lower median PFS (*p* = .004) and OS (*p* = .026) than earlier stages. Non‐serous carcinoma had shorter survival than serous (*p* < .001). Non‐carboplatin first‐line treatment (*p* = .003), platinum resistance, RD (*p* < .001), and lack of CA‐125 response (*p* = .004) were associated with reduced survival.

Subsequently, we examined PLAT‐M8's link to recurrence‐related parameters (platinum sensitivity, RECIST, CA‐125 response). PLAT‐M8 methylation correlated with platinum sensitivity post‐first‐line chemotherapy, with Class 1 more resistant and Class 2 more sensitive (*p* = .036, Figure 3C). Class 1 was predominant in progressive disease (PD, 100%), stable disease (SD, 62.5%), and partial response (PR, 54.3%) compared to complete response (CR, 40.9%) (*p* = .004, Figure 3D). While not significant, Class 2 showed a trend towards better CA‐125 response (*p* = .104, Figure 3E).

### 3.6 Prognostic performance of PLAT‐M8

**FIGURE 4:**
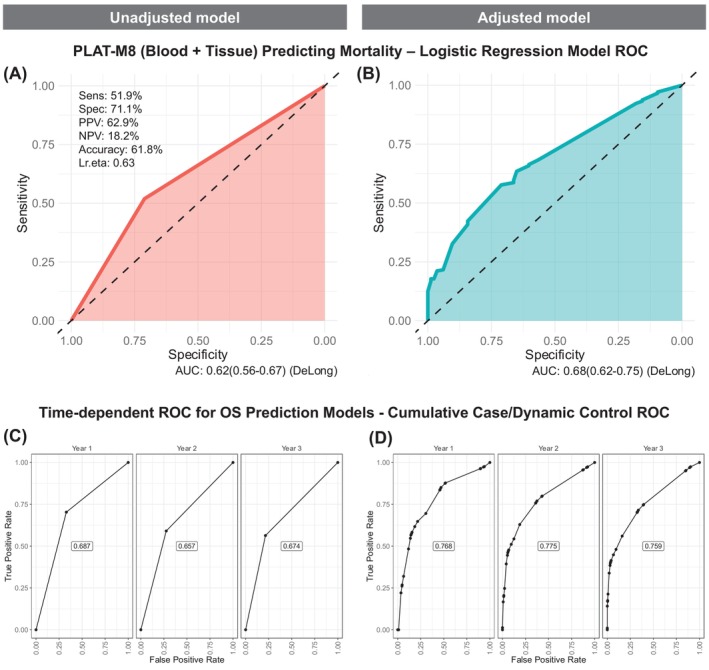
Prognostic performance of PLAT‐M8 in blood and tumour DNA for mortality risk and time‐dependent survival in relapsed cases (*n* = 291). (A) Using a univariate logistic regression model to assess mortality risk, PLAT‐M8 alone showed 51.9% sensitivity, 71.1% specificity, 62.9% positive predictive value (PPV), 18.2% negative predictive value (NPV), and 61.8% accuracy (AUC = 0.620). (B) Using a multivariate logistic regression model incorporating age at relapse, FIGO stage, tumour histology, and progression‐free survival (PFS), PLAT‐M8 showed improved mortality prediction (AUC = 0.680). (C) Using a univariate Cox‐regression model to assess time‐dependent overall survival (OS), PLAT‐M8 achieves peak discrimination in year one (AUC 0.687), declining to 0.674 by year three. (D) After adjusting for age at relapse, FIGO stage, histology, and PFS, PLAT‐M8's discrimination improves in year one (AUC 0.768), declining to 0.759 by year three.

To further measure the prognostic performance of PLAT‐M8, we analysed PLAT‐M8's ROC curves for mortality prediction using univariate and multivariate logistic regression in blood and tissue biopsy (Figure 4A,B) and blood‐only datasets (Figure S6). In univariate analysis, PLAT‐M8 had moderate sensitivity (51.9%), good specificity (71.1%), and sufficient area under the curve (AUC, 0.62). Adding clinical covariates improved AUC but remained within a sufficient range. To refine performance assessment, we applied a dynamic control approach with cumulative (Figure 4C,D) and incident case ROCs (Figure S7). The cumulative case ROC in the multivariate model showed good OS discrimination, with AUC starting at 0.768 and stabilising at 0.759 in year three, outperforming the unadjusted model.

Furthermore, we conducted a sensitivity analysis of PLAT‐M8 across different stages of cancer progression, demonstrating greater prognostic value post‐relapse, particularly in partially sensitive (PFS/PFI 6–12 months) and sensitive (PFS/PFI >12 months) patients. Sensitivity analysis by histological type also confirmed PLAT‐M8's effectiveness in both serous and non‐serous cancer (Table 2). Additional analysis was performed on ‘high‐grade serous ovarian carcinoma (HGSOC) only’, as the number of low‐grade serous ovarian carcinoma (LGSOC) cases was limited.

**TABLE 2:** Sensitivity analysis of Class 1 compared with Class 2 of PLAT‐M8 in different progression times and histological subtypes in assessing survival after first relapse.

Subgroups	Number at risk		Log‐rank, *p*‐value	uHR (95% CI)	Coxph, *p*‐value	aHR (95% CI)	Coxph, *p*‐value
	Class 2	Class 1					
Progression time							
PFS <6 months	5	20	.317	1.87 (0.54–6.49)	.325	1.93 (0.49–7.61)	.350
PFS 6–12 months	30	56	**.005**	2.08 (1.24–3.49)	.**006**	2.63 (1.46–4.75)	.**001**
PFS >12 months	124	56	**.008**	1.67 (1.13–2.45)	.**009**	1.61 (1.08–2.40)	.**019**
Histological subtype							
Serous carcinoma	119	87	**<.001**	2.12 (1.51–2.98)	**<.001**	1.81 (1.27–2.58)	**.001**
HGSOC only	77	57	**<.001**	2.11 (1.35–3.31)	**.001**	1.89 (1.19–3.02)	**.007**
Non‐serous carcinoma	40	45	**<.001**	2.56 (1.56–4.19)	**<.001**	2.19 (1.21–3.95)	**.009**

*Note*: Adjusted models included age, FIGO stage, histological group, and PFS time. The bold values indicate significant *p*‐values.

Abbreviations: 95% CI, 95% confidence interval; aHR, adjusted hazard ratio; HGSOC, high grade serous ovarian carcinoma; PFS, progression‐free survival; uHR, unadjusted hazard ratio.

## 4 DISCUSSION

### 4.1 Key messages

This study validates the prognostic role of the methylation‐based biomarker ‘PLAT‐M8’ in relapsed OC patients, demonstrating strong survival risk prediction in the first year and sustained performance over time. Class 1 is linked to poorer prognosis, as confirmed in three published datasets (ScoTROC‐1D, ScoTROC‐1V, and OCTIPS) and two independent validation cohorts (BriTROC‐1 and OV04). A combined analysis and meta‐cohort reinforced its reliability. PLAT‐M8 also stratified survival based on second‐line treatment but lacked early prognostic value pre‐relapse in the HH cohort. Given challenges in obtaining relapse tissue samples, blood‐based DNA methylation markers offer a promising alternative due to their high specificity and minimal sample requirements.

### 4.2 PLAT‐M8 in comparison with clinicopathological characteristics and mortality status

In our five relapsed cohorts, the median diagnosis age (61 years) and relapse age (62 years) align with global data, while median PFS (16.57 months) is slightly shorter than the reported 18–24 months.^13^ Age was linked to the epigenetic signature, suggesting tumourigenesis‐related epigenetic ageing. Bivariate analysis associated PLAT‐M8 with diagnosis age but not relapse age, suggesting ageing may contribute to observed epigenetic changes long before recurrence. Additionally, age differences between presentation and relapse within a shorter time frame (<3 years) were unlikely to cause additional methylation changes based on previous research.^14^ In our multivariate analysis of factors associated with PLAT‐M8 and mortality, we used age at relapse for practical reasons during hospital visits. Epigenetic ageing is known to drive cancer recurrence via stochastic DNA methylation, tumour suppressor disruption, and immunosenescence, linking age‐related epigenetic dysregulation to chemoresistance and OS.^15^

Our research found that early‐stage cases had a lower relapse rate (11%) than advanced stages (89%), consistent with recurrence rates of 10%–50% in early stages^16^ and over 80% in advanced stages.^17^ FIGO stage correlates with recurrence, reflecting disease aggressiveness and influencing treatment complexity, thereby increasing the risk of RD, a key relapse driver.^18^ Disease stage has also been linked to DNA methylation changes in OC progression.^19^ We found that PLAT‐M8 levels at relapse (*p* = .038) associate with clinical staging, with Class 1 (hypomethylation) in later stages and Class 2 (hypermethylation) in earlier stages, though significance diminishes after adjustment. Abnormal DNA methylation, particularly hypomethylation, is an early event in tumour initiation and is linked to chromosome instability, increased aggressiveness, and decreased survival.^20^ A previous study revealed that FIGO staging is an independent prognostic factor for relapsed OC, reflecting disease aggressiveness, spread potential, and impact on survival.^21^

Despite DNA methylation's role in epithelial OC subtypes,^22^ our analysis found no significant link between histological subtype or tumour grade and PLAT‐M8, possibly due to unequal proportions in tumour histology and grade. However, evidence suggests histotype‐specific hypermethylation linked to carcinogenic processes, immune pathways, or precursor tissue.^22^ Non‐serous carcinomas (e.g., mucinous, clear cell/CCC, endometrioid) showed poorer survival than serous carcinoma, likely due to lower chemotherapy responsiveness and limited targeted therapy. CCC is linked to higher progression rates, and mucinous cancer has lower first‐line platinum response. While early‐stage mucinous and clear cell cases have better outcomes, advanced‐stage cases fare as poorly as or worse than HGSOC.^23^ In addition, nearly 90% of our patients were diagnosed at an advanced stage, contributing to poor outcomes. Differences in chemotherapy regimens, histological compositions, and cohort timing (e.g., older ScoTROC/OCTIPS vs. recent BriTROC‐1/OV04) may also influence different survival. Further research should test this biomarker in other subtypes, such as LGSOC.

In OC, RD status after debulking surgery is a key prognostic factor for OS and PFS, indicating adverse tumour biology, severe dissemination, and progression.^18^ Our analysis found no correlation between debulking type and methylation status, but RD was linked to higher mortality. Achieving optimal debulking improves median OS by 5.5% and cytoreduction rates by 10%.^18^ In our study, 60.1% of patients had RD, likely contributing to recurrence. Bivariate analysis linked RD with hypomethylation status of PLAT‐M8 (Class 1), suggesting a connection to methylation changes. This aligns with studies linking suboptimal surgery to *SPARC* hypomethylation, increasing gene expression and invasiveness.^24^ RD may reflect unique methylation patterns driving treatment resistance,^25^ supporting PLAT‐M8 as a potential biomarker for RD detection, treatment response, and chemotherapy‐induced methylation changes. Further analysis revealed PLAT‐M8's intersection with RECIST response and epigenetic methylation in OC. While prior studies examined ctDNA‐based RECIST biomarkers, they lacked methylation analysis and focused on colorectal cancer, not OC.^26^ PLAT‐M8 offers novel insights into relapsed OC, integrating anatomical and epigenetic changes for personalised management. Studies in breast^27^ and cervical cancers^28^ linked methylation biomarkers with RECIST, while metastatic CRC showed RECIST‐associated hypermethylation (e.g., *TFAP2E*, linked to chemoresistance,^29^ and *NPY*, outperforming RECIST in PFS prediction).^30^ Our findings suggest PLAT‐M8, by reflecting tumour behaviour through epigenetics, may surpass conventional imaging markers in predicting recurrence and OS. Currently, no standard prognostic indicator exists for OC, particularly in relapsed cases. CA‐125, the most used biomarker, has inconsistent sensitivity (50%–62%) and limited prognostic value.^31^ It does not significantly correlate with mortality and lacks predictive power for relapse.^32^ Our analysis found no statistical link between PLAT‐M8 at relapse and CA‐125, reinforcing its limitations. PFI, often used in recurrence assessment, inaccurately classifies relapses based on time and lacks molecular insights into platinum resistance.

In our study, PLAT‐M8, correlates with PFS in univariate and multivariate analyses, making it a more reliable prognostic biomarker. Prior studies associate PFS with DNA methylation, identifying loci linked to shorter PFS^33^ and an 11‐gene panel (*Bmi‐1* pathway) tied to metastasis.^34^ We found that shorter PFS strongly predicts mortality and contributes to PLAT‐M8 Class 1 status, aligning with research showing that PFS reflects genomic and epigenomic tumour changes, driving poorer survival.^35^

### 4.3 PLAT‐M8 as an epigenetic‐based prognostic biomarker

PLAT‐M8 shows strong prognostic value for survival after relapse, even without secondary cytoreductive surgery data. PLAT‐M8 Class 1, characterised by hypomethylation, aligns with studies linking hypomethylation in tumour‐initiating cells to poor OC prognosis, specifically *ATG4A* and *HIST1H2BN* hypomethylation.^36^ During validation, PLAT‐M8 significantly associated with OS post‐relapse in the ‘BriTROC‐1 + OV04’ datasets, as well as five additional datasets, confirming its effectiveness across multiple sources. In Figure 1A,B, the differences between BriTROC‐1 and OV04 reflect their distinct objectives. Notably, all cohorts except HH were not initially PLAT‐M8 focused, which may explain differences in clinical outcomes. BriTROC‐1 focused on resistance acquisition from a genomic perspective, while OV04 integrated imaging and molecular data to study tumour heterogeneity, treatment response, and ctDNA dynamics. Despite design differences, their age, PFS, and OS parameters were comparable. Combining these datasets, the HR consistently favoured Class 2 of PLAT‐M8. To determine if PLAT‐M8 can serve as an early prognostic biomarker, analysis of the HH cohort during first‐line chemotherapy shows that this biomarker is effective only during relapse. This suggests PLAT‐M8 methylation reflects platinum sensitivity post‐relapse rather than pre‐existing chemoresistance.

For further sensitivity analysis, we derived platinum sensitivity categories (i.e., PFI) from cancer progression stages (i.e., PFS), despite their different starting points: PFI starts from the last platinum treatment to progression/recurrence, while PFS begins at initial surgery. Nevertheless, Mankoo et al.^37^ found a direct correlation between PFS and PFI outcomes, supporting the use of PFS to assess platinum responsiveness when PFI data is unavailable. One of the key findings is that PLAT‐M8 is a strong prognostic factor for survival after relapse in partially sensitive cases, supporting its potential for guiding clinical decisions. Our sensitivity analysis confirmed PLAT‐M8's effectiveness in both serous and non‐serous cancers, supporting its versatility across various tumour histotypes. In the HGSOC‐only analysis (a subtype with high relapse rates and comprising 70%–90% of serous carcinomas), the results supported PLAT‐M8 as a robust prognostic biomarker across major OC subtypes.

Despite factors like PFI, patient status, and toxicity guiding treatment, the role of DNA‐methylation‐based biomarkers in decision‐making remains underexplored. Therefore, PLAT‐M8 was also validated to understand its role in stratifying survival based on the treatment choice. It was tested across different second‐line chemotherapy regimens using the BriTROC‐1 and OV04 datasets. Platinum monotherapy was effective for Class 2 biomarkers (mostly platinum‐sensitive), with survival rates comparable to Class 2 patients treated with other therapies with/without platinum. For Class 1 patients (mostly platinum‐resistant), rechallenging with other therapies (paclitaxel/docetaxel, liposomal doxorubicin, and topotecan) with/without platinum combinations showed similar or better outcomes than platinum alone, consistent with previous findings.^38^, ^39^ Platinum‐based combinations, especially in late recurrent disease, are known to provide better outcomes, suggesting that Class 1 patients may still benefit from them. This indicates that resistance based on PFI might exclude patients from benefiting from other therapies with/without platinum‐based combinations. On the other hand, PLAT‐M8 can tailor treatments to help Class 1 patients benefit from these therapies. Nevertheless, platinum‐only treatment for Class 1 patients should not be advised as it worsens survival outcomes.

Interestingly, Class 2 patients receiving multiple second‐line combinations had poorer survival than platinum monotherapy, possibly due to unpredictable toxicity or treatment discontinuation.^40^ In contrast, patients in Class 2 who received carboplatin alone showed improved PFS and OS, likely due to fewer adverse events.^41^ Compliance issues with platinum combinations may extend response duration, but adding new drugs risks increasing toxicity, costs, and treatment dropout without significant benefits.^40^ While chemotherapy combinations were effective in Class 2, their survival outcomes may not surpass monotherapy. This could be attributed to natural selection within the cohort, as patients receiving combination therapies were often in poorer condition, such as being resistant, progressive, or difficult to treat. Multidrug regimens, especially platinum with taxanes, can cause cumulative toxicities, affecting treatment adherence and survival. A meta‐analysis underscores the need for personalised treatment approaches considering patient factors and tolerability.^42^ Adding non‐cross‐resistant cytotoxic drugs to carboplatin and paclitaxel aims to enhance efficacy but is limited by cumulative side effects (e.g., cardiotoxicity and neuropathy), and no clear survival benefits.

Given these limitations of conventional chemotherapy, there is a pressing need for reliable biomarkers to guide second‐line therapy. In this study, to ensure methodological robustness and feasibility, we employed two complementary platforms: the Illumina 450 k array for discovery and pyrosequencing for validation. This cross‐platform approach highlights the reproducibility of our findings while also demonstrating the accessibility of these methods in clinical research. Looking ahead, next‐generation sequencing (NGS)–based methylation assays are becoming increasingly available and may provide broader genomic coverage at reduced costs as the technology continues to mature and is progressively integrated into healthcare systems (e.g., *NHS*). Larger prospective cohorts or randomised trials are needed to evaluate these strategies, with PLAT‐M8 potentially serving as a prognostic biomarker to optimise clinical decision‐making for second‐line therapy in relapsed OC.

### 4.4 Proposed mechanisms

Proposed mechanism of PLAT‐M8 involves MMR, recognising platinum‐induced DNA damage, leading to hypermethylation. During first‐line chemotherapy, platinum adducts stall replication in cells, leading to increased methylation. In patients with proficient MMR, these adducts are not repaired but cause replication stalling and cell death.^43^ Ultimately, increased methylation improved patient survival, classifying them as Class 2. When MMR is lost, cells increasingly bypass platinum lesions during replication, leading to greater tolerance of platinum‐induced DNA damage, and promoting resistance without significantly changing the methylation levels. This mechanism is proposed to link DNA methylation, PLAT‐M8, platinum‐induced damage, and related oncogene expression.^43^

PLAT‐M8, consisting of 8 CpGs, is linked to eight genes (*ZNF385D*, *ZPLD1*, *MAD1L1*, *SAMD12*, *ARID5B*, *DUSP6*, *PPP2R5E*, and *SBNO2*) which are associated with carcinogenesis, chemoresistance, and prognosis.^43^ For example, *DUSP6* overexpression has been linked to chemotherapy resistance in OC,^44^ while hypermethylation of the *MAD1L1* promoter influences prognosis in advanced OC and correlates with chemotherapy response.^45^ Additionally, reduced expression of *ARID5B* indicates poor prognosis in OC.^46^ Although the roles of the other genes in OC are less well studied, they have shown prognostic significance in other cancers. For example, *ZNF385D* and *ZPLD1* are prognostic in liver^47^ and breast cancer,^48^ respectively; *SAMD12* is relevant in gastric cancer prognosis,^49^ and elevated *SBNO2* levels are associated with adverse outcomes in cervical cancer.^50^ Further experimental validation is needed to confirm their involvement by assessing the impact of methylation on gene expression.

### 4.5 Strengths and limitations

This study meticulously analysed clinicopathological characteristics related to methylation‐based biomarkers in five relapsed OC cohorts. The multi‐institutional design and large cohorts enhance generalizability. The retrospective design provides access to clinical endpoints, and blood‐based samples offer practicality and less invasiveness, benefiting personalised medicine for relapsed patients. Our analysis highlights PLAT‐M8's unique prognostic value beyond standard clinical and histopathologic features. It addresses a critical gap in relapsed OC, offering prognostic clarity not provided by existing markers. Longitudinal data show that PLAT‐M8 methylation changes over time correlate with survival, offering real‐time insights for clinicians. Its non‐invasive nature makes it a promising tool for personalising treatment strategies, such as identifying patients for platinum rechallenge. These findings underscore PLAT‐M8's potential for clinical integration and further validation in prospective cohorts.

However, this study has several limitations. First, all cohorts were sourced exclusively from the United Kingdom, requiring further investigation to assess broader applicability. This is due to challenges in obtaining relapsed OC data from international trials. Second, the study predominantly includes Caucasian participants, potentially limiting insights into population‐specific methylation differences. Collaborative efforts with researchers in Asia, Africa, and Latin America could help explore population‐level variations across diverse ethnic groups. Third, the retrospective design resulted in small sample sizes for certain subgroups (e.g., older age, early‐stage FIGO, platinum‐resistant patients) and missing data for key clinical variables, including second‐line therapy and RECIST response. These analyses were restricted to patients with complete data to maintain validity. Fourth, differences in survival between cohorts reflect heterogeneous patient characteristics and recruitment at different time points, introducing era bias, which may affect comparisons across studies. Finally, while PLAT‐M8 demonstrates moderate to strong prognostic performance, its predictive accuracy for long‐term individual outcomes is influenced by subsequent treatments, disease progression, and comorbidities. Ongoing prospective studies are designed to address these limitations by collecting longitudinal, uniformly processed samples, enabling dynamic monitoring of PLAT‐M8 methylation changes and refinement of its clinical utility.

## 5 CONCLUSION

PLAT‐M8, an epigenetically driven biomarker, emerges as a potential game‐changer, offering a reliable biomarker for relapsed OC and potentially preventing ineffective chemotherapy reintroduction. This validation study reveals clinicopathological features associated with different PLAT‐M8 Classes. Class 1 indicates a hypomethylated signature, indicating poorer outcomes. This class presents as an older population at an advanced stage, resistant to treatment, and more progressive. Our findings support PLAT‐M8's potential as a valuable prognostic marker in OC at relapse, both in blood samples and tissue biopsies. This insight contributes to stratifying second‐line treatment strategies and assessing survival. Future research will focus on PLAT‐M8's role in predicting second‐line treatment response and understanding its mechanism in chemoresistance, emphasising CpG‐associated gene expression influenced by methylation. Further validation and cost‐effectiveness assessments are needed to establish PLAT‐M8's clinical position relative to existing biomarkers like CA‐125, RECIST, or PFI, with future comparisons among patients with different responses to second‐line treatment based on RECIST.

## AUTHOR CONTRIBUTIONS

**Muhammad Habiburrahman:** Conceptualization; investigation; funding acquisition; writing – original draft; methodology; visualization; writing – review and editing; software; formal analysis; project administration; data curation; resources. **Nahal Masrour:** Data curation; resources; writing – review and editing. **Naina Patel:** Data curation; resources; writing – review and editing. **Anna M. Piskorz:** Data curation; resources; writing – review and editing. **Robert Brown:** Validation; resources; writing – review and editing. **James D. Brenton:** Data curation; validation; resources; writing – review and editing. **Iain A. McNeish:** Validation; writing – review and editing; data curation; supervision; resources. **James M. Flanagan:** Conceptualization; investigation; funding acquisition; writing – original draft; methodology; validation; visualization; writing – review and editing; software; formal analysis; project administration; data curation; supervision; resources.

## CONFLICT OF INTEREST STATEMENT

The authors declare no conflicts of interest.

## ETHICS STATEMENT

Ethical approval for the collection of additional blood samples from ovarian cancer patients undergoing first‐line chemotherapy at Hammersmith Hospital was granted by the Imperial College Healthcare Tissue Bank (ICHTB) (REC no.: 12/WA/0196, project R17016, HTA license: 12275). The study also complies with prior approvals, including SCOTROC‐1, BriTROC‐1, OCTIPS, and OV04, each approved by the relevant ethics committees. All patients provided informed consent to participate in the study and for publication, with identity details anonymised in accordance with the principles of the Declaration of Helsinki.

## Supporting information


**Data S1.** Supporting Information.

## Data Availability

The data that support the findings of this study are available from the corresponding author upon reasonable request.
