# Supplementary material for: Clinical validation of a DNA methylation biomarker associated with overall survival of relapsed ovarian cancer patients
Source: Int J Cancer. 2025 Nov 1;158(7):1821–35. doi: 10.1002/ijc.70217 (PMC12875174; doi:10.1002/ijc.70217)
Supplement: Supplementary file 1 — Data S1. Supporting Information. [file IJC-158-1821-s001.pdf]

Clinical validation of a DNA methylation biomarker associated with overall survival of relapsed ovarian cancer patients

Muhammad Habiburrahman, Nahal Masrour, Naina Patel, Anna M Piskorz, Robert Brown, James D Brenton, Iain A McNeish, and James M Flanagan

Appendix Supplementary data

Table of contents

Table of contents **1**
Supplementary Method **2**
Table S1: Distribution of PLAT-M8 classification in different datasets of cohorts **8**
Table S2: Baseline characteristics of six cohorts **8**
Table S3: Clinicopathological features by mortality status **9**
Table S4: Clinicopathological features by biomarker class **11**
Table S5: Multivariate logistic regression analysis of factors associated with Class-1 .... **13**
Table S6: Second-line chemotherapy regimens by class and biomarker status **14**
Table S7: Multivariate Cox regression analysis associating OS with biomarker in HH cohort **14**
Table S8: Multivariate Cox regression analysis associating OS with biomarker in five cohorts. **14**
Table S9: Time-dependent ROC curves for survival prediction using DNA methylation **15**
Table S10: Median survival times and survival differences in relapsed ovarian cancer. **15**
Figure S1: Study flowchart **17**
Figure S2: Quality control and methylation analysis of blood from relapsed cases **18**
Figure S3: Blood methylation percentage by relapse status and PLAT-M8 class **19**
Figure S4: PLAT-M8 methylation not prognostic during first-line chemotherapy **20**
Figure S5: Validation of PLAT-M8 across five cohorts with harmonised data **21**
Figure S6: Performance of PLAT-M8 in blood for mortality and time-dependent survival **22**
Figure S7: Time-dependent ROC curves for survival prediction **23**
References **24**

Supplementary methods

Data source and samples

This ovarian cancer (OC) validation study integrated biomarker data from Flanagan et al.[1]—including retrospective ScoTROC 1 Discovery (1D), ScoTROC 1 Validation (1V), and OCTIPS subsets—with three additional cohorts: retrospective BriTROC-1, OV04[2], and the prospective Hammersmith Hospital (HH) prospective study (2007–2014). All studies included blood DNA, except OCTIPS that provided tissue DNA. The reason for using subsets from Flanagan et al.[1] is that they have not comprehensively analysed the clinicopathological characteristics of patients. By combining this data with evidence from the BriTROC 1, OV04, and HH cohorts, we aimed to draw conclusions about how these clinicopathological factors are associated with survival and need to be adjusted. Additionally, we examined how these factors relate to biomarker classes, noting that Class 1 is associated with poorer outcomes and traits, while Class 2 shows the opposite. Another reason used the previous subsets data is also to show the evidence so far of this biomarker tested in multiple cohorts and being a foundation to move further in prospective cohort and clinical trial. ScoTROC 1 (1998–2000) is a phase III international prospective randomised trial comparing carboplatin-docetaxel with carboplatin-paclitaxel for stage Ic–IV ovarian and/or peritoneal cancers.[3-7] OCTIPS (1985–2013) is a consortium study funded by the European Community's Seventh Framework Programme.[8-13] BriTROC 1 (2013–2017), supported by Ovarian Cancer Action (OCA) and sponsored by NHS Greater Glasgow and Clyde.[14-18] The OV04 study (2010–2018), backed by the Cancer Research United Kingdom Cambridge Cancer Centre and the Mark Foundation Institute for Integrated Cancer Medicine, aims to explore factors influencing chemotherapy effectiveness for OC.[2, 17, 19-21]

DNA was obtained from whole blood samples of 141 out of 1,077 patients in the ScoTROC 1 phase III.[3] It is noteworthy that the ScoTROC 1 datasets have been transformed into ScoTROC 1V and ScoTROC 1D subsets.[1] Additionally, blood-based DNA samples were collected from 47 out of 220 patients in the BriTROC 1[14] and 57 out of 85 patients in OV04 study.[2] All of the aforementioned cohorts provided DNA from blood samples, except for the OCTIPS study, which provided DNA from 46 tumour tissue samples out of 131 patients. This study was first published in 2013.[22] In total, DNA (from both blood and tissue) was extracted from the samples of 291 patients with OC at relapse, based on the prior evidence that methylation patterns in blood and tissue are similar.[1] We also extracted additional samples from the HH cohorts, which were collected during first-line chemotherapy prior to relapse on cycles 3, 4, and 6, totaling 153 blood samples from 100 patients. All patients provided written informed consent for sample collection, and the study received approval from the appropriate ethical review boards.

Patients characteristics

In total, we analysed data from 391 patients (291 with relapsed cases and 100 with primary diagnoses) with epithelial OC, which includes high-grade serous ovarian carcinoma/HGSOC, clear cell carcinoma/CCC, endometrioid carcinoma, and mucinous carcinoma. Cases that did not meet the inclusion criteria or had poor sample quality were excluded.

Patient data in the ScoTROC-1 datasets included women aged ≥18 with epithelial ovarian carcinoma or ovarian-type peritoneal carcinomatosis. These individuals had not undergone prior chemotherapy or radiotherapy and had completed six cycles of chemotherapy at 3-week intervals. Inclusion criteria specified International Federation of

Gynecologic Oncology (FIGO) stages IC–IV, an Eastern Cooperative Oncology Group (ECOG) performance status of 0–2, and sufficient levels of bone marrow, hepatic, and renal function.[3] For the OCTIPS study, women aged ≥18 with OC were recruited and followed through primary cytoreduction and adjuvant platinum-based chemotherapy. The study included patients who underwent both primary and recurrent OC surgery in European Gynaecologic Oncology referral centers.[10, 12]

In the BriTROC 1 datasets, eligible women were aged ≥18 years, with a life expectancy of at least three months, had recurrent histologically-proven OC, primary peritoneal carcinoma, or fallopian tube cancer of High-Grade Serous OC (HGSOC) and high-grade endometrioid subtypes. These patients had relapsed following at least one line of platinum-based chemotherapy, and those with other histological subtypes had known deleterious germline *BRCA1* or *BRCA2* mutations.[14, 18] The OV04 recruited patients with cancer of the ovary, fallopian tube, or primary peritoneal cancer, collecting blood samples before and after initiating treatment with surgery or chemotherapeutic agents.[21] Patients from Hammersmith Hospital with non-relapsed OC met the inclusion criteria except for not having experienced a relapse, undergoing first-line platinum-based chemotherapy instead.

Clinical variable descriptions

Clinicopathological characteristics were retrieved from all cohorts, as they had not been comprehensively analysed in relation to mortality and biomarker statuses in previous studies. Histological subtype and cancer stage were confirmed at the time of diagnosis. This study focuses on relapsed cases and was initially designed to be inclusive and practical for all histological subtypes of OC. However, it was not originally intended to compare non-serous tumours with serous carcinomas or low-grade with high-grade serous carcinomas. Analysis of low- and high-grade tumours was limited due to the predominance of HGSOC among our recruited patients, as this subtype is known to relapse most frequently within two years. Additionally, tumour grade classification was not possible for all cases due to missing information from several cohorts. Nevertheless, we were able to provide classifications for well-differentiated (G1), moderately differentiated (G2), and poorly/undifferentiated tumours (G3–4). Moreover, the unequal proportions of histological subtypes in clinical practice further complicate the ability to draw conclusions from the small representation of certain subtypes.

For treatment response variables, Response Evaluation Criteria in Solid Tumours (RECIST) defined complete response, partial response, stable response, and progression after first-line chemotherapy, following the RECIST response.[23] CA-125 responsive was defined as a minimum 50% decrease in CA-125 values during two weeks.[24] Progression or non-response was defined as CA-125 non-response or progression in three scenarios: First, for patients with initially high CA-125 levels that normalise, progression is indicated if CA-125 becomes at least twice the upper limit of the reference range on two occasions separated by at least one week.[24] Second, for those with persistently elevated CA-125 levels before treatment, progression is defined as CA-125 reaching at least twice the nadir value on two occasions with a one-week interval. Third, patients with CA-125 initially within the reference range must later show CA-125 levels at least twice the upper limit of the reference range on two separate occasions at least one week apart.[24]

Age category was determined by Youden index analysis from receiver operating characteristic (ROC) curve analysis related to mortality[25], and consistent with a previous European study.[26] Patient ages at presentation and relapse were categorised per decade, with 'younger patients' defined as those aged 75 years old or younger, reflecting the peak

rate of OC cases in the UK.[27] Clinical endpoints included progression-free survival (PFS) and overall survival (OS) after relapse. OS was defined as the time from recurrence diagnosis to death or censoring.[28] PFS refers to the duration starting from the initial surgical resection until either the occurrence of progression, recurrence or the last known contact date, provided the patient is alive and has not experienced a recurrence. PFS at first relapse dichotomised as >327 and ≤327 days based on the Youden index from ROC curve analysis related to mortality.[25] On the other hand, a similar parameter, platinum-free interval (PFI) is defined as the duration from the last primary platinum treatment until the onset of progression, recurrence, or the last known contact date, assuming the patient is alive and has not encountered a recurrence.[29] PFI classification was simplified into platinum-sensitive (≥6 months since last chemotherapy) or platinum-resistant (<6 months since last chemotherapy) based on prior research.[30] Follow-up time was defined as the time from initial presentation to death or censoring.[28] Patients who survived until the end of the observation period were censored at their last follow-up visit.

Methylation analysis

Eight CpG sites of PLAT-M8 is *cg05529343*, *cg12992827*, *cg16172923*, *cg07960624*, *cg25953130*, *cg13691961*, *cg01692018*, and *cg07573872*) which were previously identified.[1] The presence of methylated alleles in tumour DNA pools, but not in normal cells, indicated abnormal methylation patterns in tumours.[1] The presence of methylated alleles in tumour DNA pools, but not in normal cells, indicated abnormal methylation patterns in tumours.[1] The EZ-96 DNA Methylation Gold kit (Zymo Research, Orange, CA) was used to bisulfite convert genomic DNA following the manufacturer's instructions.

We analysed methylation data from multiple cohorts, employing various methods that were ultimately normalised to ensure consistency across analyses. The initial work by Flanagan et al.[1] utilised three subsets. The ScoTROC-1D cohort (54 relapsed cases) represented the discovery phase, where methylation was analysed using a 450K array on blood samples. The ScoTROC-1V cohort (87 relapsed cases) served as the validation phase, with methylation measured using pyrosequencing on blood samples. Additionally, the OCTIPS cohort (46 relapsed cases) was used to investigate whether the blood methylation patterns observed in the biomarker could also be detected in tissue, with analyses conducted via pyrosequencing. For further validation, new cohorts were included. The BriTROC-1 cohort (47 relapsed cases) used an Infinium HumanMethylation450 BeadChip array (“450 K array”) to analyse blood methylation. The OV04 cohort (57 relapsed cases) employed pyrosequencing for blood methylation analysis. Lastly, the HH cohort included 100 patients and 153 samples of non-relapsed cases undergoing first-line chemotherapy. This cohort, with methylation assessed via pyrosequencing, aimed to explore the biomarker's utility before relapse. Despite these varied methods, all data were normalised to ensure robust and comparable analyses across the different cohorts.

Pyrosequencing was carried out according to established protocols. Detailed methods for DNA bisulfite treatment and PCR product sequencing are described in our previous publications.[1, 31] Significance between groups was determined using the Wilcoxon signed-rank sum test. Specific primers for the eight CpG loci were designed for pyrosequencing using PyroMark software (Qiagen, Hilden, Germany), and the sequences and annealing temperatures used in this study were consistent with previous reports (See: **Table. Primers** below).[1, 31, 32] After quality control, samples not meeting quality standards were omitted. A functional normalisation method was used for normalisation, generating β and M values.[33, 34] Missing values for these values were imputed using K-nearest neighbor (*knn*) "Impute"

function. Probes with cross-hybridisation or single nucleotide polymorphisms (SNPs) were not preemptively excluded but considered for subsequent validation.[35]

Supplementary methods table. Primers for PCR and pyrosequencing used in this study for the PLAT-M8 methylation assay.[1]

No	CpG sites	Gene	Type of assay	Primer *=biotinylated	Primer sequence	Annealing temperature
1	cg05529343	*ZNF385D*	PCR	Forward	GTTTAAAGAGGTTGTGTAGGGGTTA	62.0° C
			PCR	Reverse*	CACCTAAAATATATTTCCCATTCACT	61.0° C
			Pyrosequencing		GTTGTGTAGGGGTTAAT	
2	cg12992827	*ZPLD1*	PCR	Forward	GTTAGGAATTATTAATTAGGTAGTATG	54.0° C
			PCR	Reverse*	ACCTATTAACCCAACCACTAAATT	59.5° C
			Pyrosequencing		AGGTAGTATGTTTAGAATGT	
3	cg16172923	*MAD1L1*	PCR	Forward	GAGGGGAGGTTTGAATTTT	59.4° C
			PCR	Reverse*	TAACTATACTAAAAACCTCCTAAAATAC	55.1° C
			Pyrosequencing		TGGGGTTTTTTTTGTAG	
4	cg07960624	*SAMD12*	PCR	Forward	ATGGGTAATTAGTTTTGGAAGATTTT	61.7° C
			PCR	Reverse*	ACCAAACTTATAATAAATCATCCTACTAC	58.2° C
			Pyrosequencing		GTAATTAGTTTTGGAAGATTTTTAA	
5	cg25953130	*ARID5B*	PCR	Forward	AAATGTATGGATAGAAAATAGGAAATGT	60.9° C
			PCR	Reverse*	TTCTCACAATAATTTATACTTCCTCC	59.4° C
			Pyrosequencing		AGGTATATAAAGTTGATGTTTT	
6	cg13691961	*DUSP6*	PCR	Forward	GGGTGGTTTTTAGTTTGTTGAGATT	63.9° C
			PCR	Reverse*	CCAACAACTAACCCATAAAATTAAAATT	62.7° C
			Pyrosequencing		GGTATATTGTTTGGTTGGT	
7	cg01692018	*PPP2R5E*	PCR	Forward	GTTGGGGAATTAAAGGAGAATTAAAATA	63.8° C
			PCR	Reverse*	ACTCCACACACACCAAACTAATCTA	62.5° C
			Pyrosequencing		ATTAAAGGAGAATTAAAATAATAAG	
8	cg07573872	*SBNO2*	PCR	Forward	AGGAAAGAAGTTAGGGTTTGAT	58.7° C
			PCR	Reverse*	CCACAACTTCTACATTTTCTAACACTAA	61.4° C
			Pyrosequencing		CCTAAAAAACTAAATCACCAT	

To classify the patients into two classes (Class 1, low/no methylation; Class 2, high methylation), we did not use an established cutpoint to distinguish between class 1 and class 2. Instead, classification was based on consensus clustering using the methylation scores of 8 CpG sites, following the methodology of a previous study.[1] We used a data matrix of methylation beta values for these eight selected probes for each sample, employing the "*ConsensusClusterPlus*" package in R with standard parameters: Euclidean distance, up to k = 6 groups, and n = 100 bootstrapped samples with 80% resampling. During the discovery of this biomarker in previous research, we performed univariate analysis using the Cox proportional hazards model to identify 8 probes (out of 333, FDR < 10%) associated with overall survival (time from relapse to time of death). Consensus clustering of the methylation values for these 8 probes identified two groups. The methylation scores differ across the 8 genes, but when combined into an 8-signature, the overall trend shows increased hypermethylation and decreased hypomethylation. These sites are located within different genes, contributing to the overall score. While patients may exhibit varying methylation values at individual CpG sites, the averaging process mitigates the impact of outliers, providing a more stable overall score. This approach captures the broader methylation landscape associated with tumour behavior.[1]

Statistical analysis

The association between clinicopathological factors, mortality status, and biomarker status was assessed using statistical tests such as the $\chi 2$, Fisher's exact, or non-parametric test in SPSS v29. Mann-Whitney and Kruskal-Wallis tests were used for abnormally distributed data to determine the statistical significance of differences in average age across groups.[36] Life tables and the log-rank test were used to compare median survival times and rates across cohorts. Factors such as age at relapse, FIGO stage, grade, histological subtype, first-line chemotherapy class, residual tumour after surgery, and PFS time were analysed in the multivariate logistic regression to identify factors linked with Class 1 of PLAT-M8. The percentages of PLAT-M8 Class 1 and Class 2 were also compared across cohorts using the $\chi 2$ test. We aimed to understand which factors contribute to the poorer class of PLAT-M8.[37]

In subsequent statistical analysis, we used R v4.3.1 and RStudio. The Kaplan-Meier (KM) method was used for OS in the BriTROC 1 and OV04 datasets, comparing Class-1 and Class-2 groups. The Log-rank test determined group differences.[38] In the 'BriTROC 1 + OV04 study', OS analysis compared patient groups based on their assigned second-line chemotherapy regimens. Individual survival analysis related to the biomarker was not done on the ScoTROC 1 (V and D) and OCTIPS datasets, because those data have already been published elsewhere[1], except for clinicopathological analyses. Additionally, we could not stratify survival analysis based on second-line chemotherapy for these two cohorts due to the lack of information. However, we still performed Cox proportional hazards regression analyses combining harmonised data from five subsets of prior and current validations of PLAT-M8, reflected as a hazard ratio (HR) in the KM curve. Biomarker status, along with adjusted clinicopathological factors, was included in a multivariate Cox regression model to assess OS.[37] Heterogeneity across different relapsed cohorts was not considered at this stage. To present current existing evidence of PLAT-M8 while accounting for possible heterogenity, the HR from univariate Cox regression analysis was assessed using meta-analysis. Forest plots, using a random-effects model, were generated to summarise HR for overall effect size estimates in OS analysis, along with an evaluation of heterogeneity.[39]

Sensitivity analyses of PLAT-M8 in assessing survival after relapse were conducted based on progression time—used as a surrogate for platinum sensitivity—and histological subtype (serous vs. non-serous; HGSOC [grades 3–4]. Platinum sensitivity categories (i.e., PFI) were derived using cancer progression stages measured by PFS, despite their different starting points: PFI is measured from the last platinum treatment to progression or recurrence, while PFS begins at initial surgery.[40] Nevertheless, Mankoo et al.[29] demonstrated a direct correlation between PFS and PFI outcomes, supporting the use of PFS to assess platinum responsiveness when PFI data are unavailable. Not all patients with serous carcinoma had tumour grade information, which limited the analysis (available for 145 of 206 patients; 70.4%). For histological subtype analyses, we used available data to evaluate HGSOC (134 of 138 high-grade OC cases, with 4 non-serous types). For an additional sensitivity analysis on prognostic evaluation, time-dependent survival receiver operating characteristic (ROC) curves were used to assess cumulative incidence and risk prediction.[41] Statistical significance was set at $p<0.05$.

In terms of statistical analysis, we used SPSS v29, R v4.3.1, and RStudio with the following packages: *survminer, survival, survMisc, broom, dplyr, tidyverse, lubridate, ggplot, ggpubr, ggsurvfit,* and *pROC*. To build statistical models, missing clinical variables that might be significant in univariate analysis—such as (1) first-line chemotherapy class, (2) PFI/platinum sensitivity levels, and (3) residual disease for adjusted Cox regression modeling, as well as (1) residual disease, (2) RECIST response, and (3) PFI/platinum sensitivity levels for adjusted/multivariate

logistic regression—were not subjected to multiple imputations. While multiple imputations can be useful, it is unsuitable for our study due to several challenges. Variables like first-line chemotherapy class and platinum sensitivity are influenced by patient-specific clinical decisions, making accurate prediction of missing values difficult and prone to bias. Multiple imputation assumes data are missing at random (MAR), but our missing data stems from systematic non-recording across cohorts, likely violating this assumption.[42, 43] Cohort-specific characteristics may drive the missingness, which imputation cannot adequately address. Key variables, such as PFI, residual disease status, and RECIST response, are interdependent. Imputing one based on another risks circular reasoning and undermines analytical validity. Furthermore, using imputed values in regression models could produce unreliable results if they fail to reflect true clinical scenarios. To avoid these risks, we excluded these variables from our models, focusing instead on complete data, which includes five variables in Cox regression and six in logistic regression. This approach ensures robust analysis without introducing unnecessary bias. Our decision highlights the limitations of imputation in this context and the importance of maintaining analytical integrity through alternative methods.

We acknowledge the potential risk of skewed data arising from the inclusion of previous positive-result cohorts containing blood and tissue samples where this biomarker was previously tested. To summarise the existing evidence for this biomarker across multiple cohorts and address potential bias, we conducted a meta-cohort analysis using a random-effects model along with heterogeneity tests, including Cochran's Q Test and the I^2 Statistic (Higgins' I^2). Furthermore, the OCTIPS study, which exclusively utilised tissue samples, has demonstrated that these samples exhibit the same methylation patterns as blood.

Ethical approval

Ethical approval for the additional collection of blood samples from patients with OC undergoing first-line chemotherapy from HH was granted by the Imperial College Healthcare Tissue Bank (ICHTB) Research Ethics Committee (REC no: 12/WA/0196, project application number: R17016, and ICHTB Human Tissue Authority (HTA) license: 12275) on 2nd May 2017. The study also adheres to ethical standards from prior approved studies, including: (1) ScoTROC 1: Ethical oversight provided by multiple centres[3]; (2) BriTROC 1: Approved by the Cambridge Central Research Ethics Committee, UK (No: 12/EE/0349)[14]; (3) OCTIPS: Approved by multiple local ethics committees, such as Charité-Universitätsmedizin Berlin (Germany), Medical University of Innsbruck (Austria), Katholieke Universiteit Leuven (Belgium), University of Edinburgh (UK), and other centres that joined this consortium (Nos: EK207/2003, ML2524, 05/Q0406/178, EK130113, 06/S1101/16)[10, 13]; (4) OV04: Approved by the National Research Ethics Service (NRES) Committee East of England - Cambridge Central, UK (No: 07/Q0106/63) for Addenbrooke's Hospital, Cambridge, as well as the Cambridge Central Research Ethics Committee (No: 03/018).[44] The reporting and writing of this research followed the REporting recommendations for tumour MARKer prognostic studies (REMARK)[45] and the Strengthening the Reporting of Observational Studies in Epidemiology (STROBE) guidelines.[46]

Public involvement and role of funders

Patients provided feedback on the study design and emphasised its significance for improving OC treatment. The funders had no role in the study's design, data collection, analysis, interpretation, or report writing.

Supplementary tables

Table S1. Distribution of PLAT-M8 classification in different datasets of cohorts, according to clustering analysis consensus (n = 391 patients, 444 samples).

Cohorts	Biomarker status[a]				Total		p-value
	Class 2		Class 1				
	n	%	n	%	n	%	
ScoTROC 1D	25	46.3	29	53.7	54	12.2	0.013[a]
ScoTROC 1V	43	49.4	44	50.6	87	19.6	
OCTIPS	28	60.9	18	39.1	46	10.4	
BriTROC 1	31	66.0	16	34.0	47	10.6	
OV04	32	56.1	25	43.9	57	12.8	
HH sample on cycles 3&4[b]	69	67.0	34	33.0	103	23.2	
HH sample on cycle 6[b]	20	40.0	30	60.0	50	11.3	
Total relapse cases	159	54.6	132	45.4	291		
Total non-relapse samples	89	58.2	64	41.8	153		
Overall cases	248	55.9	196	44.1	444		

The percentage '%' values in the biomarker status column represent row percentages, meanwhile in total column represent column percentages. [a]Chi-square test. [b]When the analysis focuses on 100 patients, excluding cycle-specific data, the number of patients in each biomarker status is Class 1 (n = 33) and Class 2 (n = 67)

Table S2. Baseline characteristics of patients in the six included cohorts (n = 391).

Baseline characteristics	Median (IQR) or median of survival time (95%CI) across different cohorts[a]							
	Relapse							Non-relapse
	ScoTROC 1D	ScoTROC 1V	OCTIPS	BriTROC 1	OV04	Overall relapsed cases[b]	p-value[b]	HH
	N = 54 (13.81%)	N = 87 (22.25%)	N = 46 (11.76%)	N = 47 (12.02%)	N = 57 (14.58%)			N = 100[h] (25.58%)
Age at diagnosis (years), n = 391	60.5 (52.0-65.0)	60.0 (53.0-66.0)	60.0 (53.0-66.0)	63.0 (53.0-70.0	66.0 (55.5-72.0)	61.0 (53.0-67.0)	**0.004[f]**	63.5 (54.0-70.0)
Age at relapse (years), n = 291	61.5 (53.0-65.2)	61.0 (54.0-67.0)	61.0 (54.0-67.0)	65.0 (55.0-73.0)	69.0 (57.0-74.0)	62.0 (54.0-69.0)	**0.003[f]**	-
Median PFS (months), n = 291	10.9 (9.9-11.9)	12.0 (9.8-14.2)	28.8 (18.4-39.2)	24.8 (16.4-33.3)	22.0 (15.6-28.4)	16.6 (14.2-19.0)	**<0.001[f]**	-
Median OS[c] (months), n = 391	9.1 (4.0-14.3)	13.6 (6.2-21.0)	50.1 (35.9-64.4)	18.4 (10.8-26.0)	21.9 (16.6-27.2)	19.9 (15.9-23.9)	**<0.001[g]**	26.1 (18.8-33.4)
2-year OS rate[d], n = 391	18%	22%	65%	35%	31%	32%	**<0.001[g]**	40%
Median follow up from initial diagnosis (months), n = 391[e]	120.3 (37.5-203.1)	122.5 (50.5-194.5)	110.2 (79.2-141.2)	67.9 (64.7-71.1)	59.9 (39.7-80.0)	80.7 (62.2-99.2)	**<0.001[g]**	63.3 (52.9-73.6)
Median follow up from relapse timepoint (months), n = 291[e]	86.4 (27.6-145.2)	72.7 (44.2-101.1)	73.2 (57.0-89.3)	36.7 (31.0-42.5)	30.6 (7.4-53.9)	48.1 (29.1-67.1)	**<0.001[g]**	N/A

[a]Data was presented as median (IQR) due to non-normal distribution in some cohorts, while others exhibited normal distribution, prompting homogenization; Survival time: Median (95% CI); and the percentage '%' values represent row percentages; [b]Only measured the differences of characteristics across the relapsed cases; [c]OS in five cohorts measures the time from the first relapse to death or loss to follow-up (LTFU). For survivors, OS represents the time to their last follow-up (censored time). In the Hammersmith Hospital study (non-relapse cohort), OS specifically tracks the time from the last registered chemotherapy to death or LTFU; [d]The survival rate was calculated based on life tables, following the cumulative proportion surviving at the end of the interval (5 years); [e]Median follow-up was calculated using reverse Kaplan Meier from diagnosis before relapse; [f]Kruskal-Wallis test; [g]Log rank test;

[h]Selected only the first sample from each patient (if there is more than 1 sample given) to avoid duplications in calculating endpoints for the 100 patients as individuals; ***Abbreviations: BriTROC 1**, British translational research ovarian cancer collaborative 1; **HH,** Hammersmith Hospital; **OCTIPS,** Ovarian cancer therapy innovative models prolong survival; **OS,** Overall survival after relapse; **OV04,** Ovarian cancer clinical trial study 4th edition; **PFS,** progression-free survival; **SCOTROC 1,** Scottish randomised trial in ovarian cancer (D, discovery and V, validation).*

Table S3. Clinicopathological features in five cohorts with relapsed ovarian cancer cases (n=291) and at Hammersmith Hospital with non-relapsed cases (n=100) in relation to mortality status.

Clinicopathological features	Five cohorts							Hammersmith Hospital cohort						
	Mortality status				Total		p-value	Mortality status				Total		p-value
	Alive		Death					Alive		Death				
	n	%	n	%	n	%		n	%	n	%	n	%	
**Age at diagnosis (years)**														
21-30	0	0	4	100	4	1.4	0.112[a]	0	0	1	100	1	1.0	0.916[a]
31-40	1	16.7	5	83.3	6	2.1		2	40.0	3	60.0	5	5.0	
41-50	13	29.5	31	70.5	44	15.1		3	23.1	10	76.9	13	13.0	
51-60	22	25.3	65	74.7	87	29.9		16	66.7	8	33.3	24	24.0	
60-70	28	26.9	76	73.1	104	35.7		11	32.4	23	67.6	34	34.0	
>70	19	41.3	27	58.7	46	15.8		10	43.5	13	56.5	23	23.0	
**Age at relapse (years)**														
21-30	0	0	3	100	3	1.0	0.089[a]							
31-40	1	25.0	3	75.0	4	1.4								
41-50	10	28.6	25	71.4	35	12.0								
51-60	23	26.1	65	73.9	88	30.2								
60-70	24	23.3	79	76.7	103	35.4								
>70	25	43.1	33	56.9	58	19.9								
**Age at diagnosis category (years)**														
Younger (≤75)	74	27.6	194	72.4	268	92.1	0.240[b]	39	43.8	50	56.2	89	89.0	0.350[b]
Elder >75)	9	35.3	14	64.7	23	7.9		3	27.3	8	72.7	11	11.0	
**Age at relapse category (years)**														
Younger (≤75)	71	27.6	186	72.4	257	88.3	0.352[b]							
Elder >75)	12	39.1	22	60.9	34	11.7								
**FIGO stage**														
I	9	64.3	5	35.7	14	4.8	**0.023[b]**	4	50.0	4	50.0	8	8.0	0.716[a]
II	5	27.8	13	72.2	18	6.2		6	54.5	5	45.5	11	11.0	
III	55	27.5	145	72.5	200	68.7		22	37.3	37	62.7	59	59.0	
IV	14	23.7	45	76.3	59	20.3		10	45.5	12	54.5	22	22.0	
**FIGO stage degree**														
Early (I-II)	14	43.8	18	56.3	32	11.0	**0.043[b]**	10	52.6	9	47.4	19	19.0	0.297[b]
Advanced (III-IV)	69	26.6	190	73.4	259	89.0		32	39.5	49	60.5	81	81.0	
**Histological subtypes**														
Serous carcinoma	70	34.0	136	66.0	206	70.8	**0.004[a]**	33	42.9	44	57.1	77	77.0	0.827[a]
Adenocarcinoma NOS	1	4.0	24	96.0	25	8.6		0	0	0	0	0	0	
Papillary adenocarcinoma	3	14.3	18	85.7	21	7.2		0	0	0	0	0	0	
Mucinous adenocarcinoma	1	25.0	3	75.0	4	1.4		0	0	1	100	1	1.0	
Endometrioid carcinoma	6	33.3	12	66.7	18	6.2		1	33.3	2	66.7	3	3.0	
Clear cell carcinoma	1	20.0	4	80.0	5	1.7		2	33.3	4	66.7	6	6.0	
Carcinosarcoma	0	0	0	0	0	0		1	50.0	1	50.0	2	2.0	
MCOA histological type	0	0	3	100	3	1.0		3	50.0	3	50.0	6	6.0	
Other ovarian malignancies	1	11.1	8	88.9	9	3.1		2	40.0	3	60.0	5	5.0	
**Histological group**														
Serous carcinoma	70	34.0	136	66.0	206	70.8	**0.001[b]**	33	42.9	44	57.1	77	77.0	0.751[b]
Non-serous carcinoma	13	15.3	72	84.7	85	29.2		9	39.1	14	60.9	23	23.0	
**Tumour grade**														
Well-differentiated (G1)	0	0	0	0	0	0	0.760[c]	2	50.0	2	50.0	4	4.2	0.979[a]
Moderately differentiated (G2)	5	45.5	6	54.5	11	7.4		6	40.0	9	60.0	15	15.6	

Poorly/undifferentiated (G3-4)	56	40.6	82	59.4	138	92.6		33	42.9	44	57.1	77	80.2	
Missing data					142							4		
**First-line chemotherapy**														
Plat monotherapy	17	35.4	31	64.6	48	21.0	0.174[a]	9	36.0	16	64.0	25	25.0	0.539[a]
Plat + taxane	36	20.5	140	79.5	176	76.9		30	44.1	38	55.9	68	68.0	
Plat + TopII inhibitor	0	0	0	0	0	0		1	100	0	0	1	1.0	
Plat + ACs	0	0	0	0	0	0		0	0	1	100	1	1.0	
Plat + alkylating agents	1	100	0	0	1	0.4		0	0	0	0	0	0	
Plat + taxane + STKi	1	50.0	1	50.0	2	0.9		0	0	0	0	0	0	
Plat + taxane + EGFR-TKi	1	50.0	1	50.0	2	0.9		0	0	0	0	0	0	
Plat + taxane + anti-VEGF	0	0	0	0	0	0		1	33.3	2	66.7	3	3.0	
Plat + taxane + TopI inhibitor	0	0	0	0	0	0		0	0	1	100	1	1.0	
Plat + taxane + ACs	0	0	0	0	0	0		1	100	0	0	1	1.0	
Missing data					62									
**First-line chemotherapy class**														
Cp monotherapy	17	35.4	31	64.6	48	21.0	**0.047[b]**	9	36.0	16	64.0	25	25.0	0.483[b]
Cp with other therapies	39	21.5	142	78.5	181	79.0		33	44.0	42	56.0	75	75.0	
Missing data					62									
**Platinum sensitivity level**														
Sensitive	17	42.5	23	57.5	40	85.1	**0.039[c]**							
Resistant	0	0	7	100	7	14.9								
Missing data					244									
**ECOG performance**														
0	12	22.6	41	77.4	53	37.6	0.200[b]							
1	8	11.1	64	88.9	72	51.1								
2	2	12.5	14	87.5	16	11.3								
Missing data					150									
**Surgical type**														
Interval debulking	23	44.2	29	55.8	52	91.2	0.385[c]							
Primary debulking	1	20.0	4	80.0	5	8.8								
Missing data					234									
**Residual disease**														
No residual disease	30	33.0	61	67.0	91	39.9	**0.033[b]**							
Any residual disease	28	20.4	109	79.6	137	60.1								
Missing data					63									
**RECIST response**														
Complete response	20	30.3	46	69.7	66	50.0	0.149[b]	27	57.4	19	42.6	46	47.9	**0.002[b]**
Partial response	4	11.4	31	88.6	35	26.5		5	20.8	20	79.2	25	26.0	
Stable response	4	16.7	20	83.3	24	18.2		5	31.3	11	68.8	16	16.7	
Progressive disease	2	28.6	5	71.4	7	5.3		1	11.1	8	88.9	9	9.4	
Missing data					159								4	
**CA-125 response**														
Response (decrease)	13	7.4	81	92.6	94	77.7	0.517[c]							
No response (stable/increase)	2	13.8	25	86.2	27	22.3								
Missing data					170									
**Second-line chemotherapy**														
Plat monotherapy	11	44.0	14	56.0	25	17.9	0.185[a]							
Taxanes monotherapy	4	66.7	2	33.3	6	4.3								
TopI inhibitor monotherapy	0	0	4	100	4	2.9								
ACs monotherapy	2	100	0	0	2	1.4								
Plat + taxanes	19	38.8	30	61.2	49	35.0								
Plat + antimetabolites	8	40.0	12	60.0	20	14.3								
Plat + TopI inhibitor	0	0	3	100	3	2.1								
Plat + ACs	9	39.1	14	60.9	23	16.4								
Taxane + ACs	0	0	1	100	1	0.7								
Plat + taxanes + anti-VEGF	0	0	3	100	3	2.1								
Plat + taxanes + ACs	1	50.0	1	50.0	2	1.4								

Plat + antimetabolites + anti-VEGF	0	0	2	100	2	1.4								
Missing data					151									
**Second-line chemotherapy class**														
Cp monotherapy	11	44.0	14	56.0	25	17.9	0.538[b]							
Other regiments +/- Cp	43	37.4	72	62.6	115	82.1								
Missing data					151									
**PFS time for first relapse (days)**														
>327	68	32.9	139	67.1	207	71.1	**0.010[b]**							
≤327	15	17.9	69	82.1	84	28.9								
**Biomarker status**														
Class 2	59	37.1	100	62.9	159	54.6	**<0.001[b]**	31	46.3	36	53.7	67	67.0	0.218[a]
Class 1	24	18.2	108	81.8	132	45.4		11	33.3	22	66.7	33	33.0	
**Chemo + biomarker status**														
Class 2, Cp only	9	69.2	4	30.8	13	12.5	0.556[a]							
Class 2, Other regiments +/- Cp	19	38.0	31	62.0	50	48.1								
Class 1, Cp only	1	16.7	5	83.3	6	5.8								
Class 1, Other regiments +/- Cp	12	34.3	23	65.7	35	33.7								
Missing data					187									

[a]Mann-Whitney; [b]Chi-Square; [c]Fisher's Exact test. The percentage '%' values in each group represent row percentages; meanwhile, the percentage '%' values in the total represent column percentages. ***Abbreviation: ACs,*** *anthracyclines;* ***Cp,*** *carboplatin;* ***ECOG,*** *Eastern cooperative oncology group;* ***EGFR-TKi,*** *epidermal growth factor receptor tyrosine kinase inhibitor;* ***FIGO;*** *International federation of gynecology and obstetrics;* ***MCOA,*** *Mixed cell ovarian adenocarcinoma;* ***NOS,*** *non-specific;* ***Plat,*** *platinum;* ***RECIST,*** *Response evaluation criteria in solid tumors;* ***STKi,*** *serine-threonine kinase inhibitors;* ***TopII,*** *topoisomerase II;* ***VEGF,*** *Vascular endothelial growth factor.* ***Notes:*** *+/- Cp' means with or without carboplatin since some patients did not receive Carboplatin as their primary therapy, and 'other' means other regimens of chemotherapy beside carboplatin. Taxane (e.g., paclitaxel and docetaxel), TopII inhibitor (e.g., etoposide), ACs (e.g., liposomal doxorubicin and epirubicin), Alkylating agents (e.g., cyclophosphamide), STKi (e.g., enzastaurin), EGFR-TKi (e.g., erlotinib and sorafenib), anti-VEGF (e.g., bevacizumab), TopI inhibitor (e.g., topotecan), Antimetabolites (e.g., gemcitabine)*

Table S4. Clinicopathological features in five cohorts with relapsed ovarian cancer cases (n = 291) and at Hammersmith Hospital with non-relapsed cases (n = 100) in relation to biomarker status (Class 1 and Class 2).

Clinicopathological features	Five cohorts							Hammersmith Hospital cohort						
	Biomarker status				Total		p-value	Biomarker status				Total		p-value
	Class 2		Class 1					Class 2		Class 1				
	n	%	n	%	n	%		n	%	n	%	n	%	
**Age at diagnosis (years)**														
21-30	3	75.0	1	25.0	4	1.4	**0.044[a]**	0	0	1	100	1	1.0	0.624[a]
31-40	5	83.3	1	16.7	6	2.1		3	60.0	2	40.0	5	5.0	
41-50	26	59.1	18	40.9	44	15.1		9	69.2	4	30.8	13	13.0	
51-60	52	59.8	35	40.2	87	29.9		17	70.8	13	29.2	24	24.0	
60-70	50	48.1	54	51.9	104	35.7		21	61.8	13	38.2	34	34.0	
>70	23	50.0	23	50.0	46	15.8		17	73.9	13	26.1	23	23.0	
**Age at relapse (years)**														
21-30	2	66.7	1	33.3	3	1.0	0.146[a]							
31-40	3	75.0	1	25.0	4	1.4								
41-50	22	62.9	13	37.1	35	12.0								
51-60	51	58.0	37	42.0	88	30.2								
60-70	50	48.5	53	51.5	103	35.4								
>70	31	53.4	27	46.6	58	19.9								
**Age at diagnosis (years)**														
Younger (≤75)	152	56.7	116	43.3	268	92.1	**0.015[b]**	59	66.3	30	33.7	89	89.0	>0.999[c]
Elder (>75)	7	30.4	16	69.6	23	7.9		8	72.7	3	27.3	11	11.0	
**Age at relapse (years)**														
Younger (≤75)	143	55.6	114	44.4	257	88.3	0.345[b]							
Elder (>75)	16	47.1	18	52.9	34	11.7								
**FIGO stage**														

I	9	64.3	5	35.7	14	4.8	0.129[b]	7	87.5	1	12.5	8	8.1	0.300[a]
II	14	77.8	4	22.2	18	6.2		8	72.7	3	27.3	11	11.0	
III	108	54.0	92	46.0	200	68.7		38	64.4	21	35.6	59	59.0	
IV	28	47.5	31	52.5	59	20.3		14	63.6	8	36.4	22	22.0	
**FIGO stage degree**														
Early (I-II)	23	71.9	9	28.1	32	11.0	**0.038[b]**	15	78.9	4	21.1	19	19.0	0.218[b]
Advanced (III-IV)	136	52.5	123	47.5	259	89.0		52	64.2	29	35.8	81	81.0	
**Histological subtypes**														
Serous carcinoma	119	57.8	93	42.2	206	70.8	0.166[a]	52	67.5	25	75.8	77	77.0	0.925[a]
Adenocarcinoma NOS	6	24.0	19	76.0	25	8.6		1	100	0	0	1	1.0	
Papillary adenocarcinoma	16	76.2	5	23.8	21	7.2		0	0	0	0	0	0	
Mucinous adenocarcinoma	1	25.0	3	75.0	4	1.4		0	0	0	0	0	0	
Endometrioid carcinoma	9	50.0	9	50.0	18	6.2		2	66.7	1	33.3	3	3.0	
Clear cell carcinoma	1	20.0	4	80.0	5	1.7		2	33.3	4	66.7	6	6.0	
Carcinosarcoma	0	0	0	0	0	0		2	100	0	0	2	2.0	
MCOA histological type	2	66.7	1	33.3	3	1.0		4	66.7	2	33.3	6	6.0	
Other ovarian malignancies	5	55.6	4	44.4	9	3.1		4	80.0	1	20.0	5	5.0	
**Histological group**														
Serous carcinoma	119	57.8	87	42.2	206	70.8	0.095[b]	52	67.5	25	32.5	77	77.0	0.836[b]
Non-serous carcinoma	40	47.1	45	52.9	85	29.2		15	65.2	8	34.8	23	23.0	
**Tumour grade**														
Well-differentiated (G1)	0	0	0	0	0	0	0.753[c]	4	100	0	0	4	4.2	0.063[a]
Moderately differentiated (G2)	6	54.5	5	45.5	11	7.4		12	80.0	3	20.0	15	15.6	
Poorly/undifferentiated (G3-4)	84	60.9	54	39.1	138	92.6		48	62.3	29	37.7	77	80.2	
Missing data					142							4		
**First-line chemotherapy**														
Plat monotherapy	31	64.6	17	35.4	48	21.0	0.118[a]	19	76.0	6	24.0	25	25.0	0.121[a]
Plat + taxane	91	51.7	85	48.3	176	76.9		45	66.2	23	33.8	68	68.0	
Plat + TopII inhibitor	0	0	0	0	0	0		1	100	0	0	1	1.0	
Plat + ACs	0	0	0	0	0	0		1	100	0	0	1	1.0	
Plat + alkylating agents	1	100	0	0	1	0.4		0	0	0	0	0	0	
Plat + taxane + STKi	0	0	2	100	2	0.9		0	0	0	0	0	0	
Plat + taxane + EGFR-TKi	2	100	0	0	2	0.9		0	0	0	0	0	0	
Plat + taxane + anti-VEGF	0	0	0	0	0	0		1	33.3	2	66.7	3	3.0	
Plat + taxane + TopI inhibitor	0	0	0	0	0	0		0	0	1	100	1	1.0	
Plat + taxane + ACs	0	0	0	0	0	0		0	0	1	100	1	1.0	
Missing data					62									
**First-line chemotherapy**														
Cp monotherapy	31	64.6	17	35.4	48	21.0	0.118[b]	19	76.0	6	24.0	25	25.0	0.269[b]
Cp with other therapies	94	51.9	87	48.1	181	79.0		48	64.0	27	36.0	75	75.0	
Missing data					62									
**Platinum sensitivity**														
Sensitive	29	72.5	11	27.5	40	85.1	**0.036[c]**							
Resistant	2	28.6	5	71.4	7	14.9								
Missing data					244									
**ECOG performance**														
0	29	54.7	24	45.3	53	37.6	0. 407[b]							
1	33	45.8	39	54.2	72	51.1								
2	6	37.5	10	62.5	16	11.3								
Missing data					150									
**Surgical type**														
Interval debulking	28	53.8	24	46.2	52	91.2	0.372[c]							
Primary debulking	4	80.0	1	20.0	5	8.8								
Missing data					234									
**Residual disease**														
No residual disease	57	62.6	34	37.4	91	39.9	**0.024[b]**							
Any residual disease	65	47.4	72	52.6	137	60.1								
Missing data					63									

**RECIST response**														
Complete response	39	59.1	27	40.9	66	50.0	**0.004[a]**	33	70.2	14	29.8	47	49.0	0.797[b]
Partial response	16	45.7	19	54.3	35	26.5		15	62.5	9	37.5	24	25.0	
Stable response	9	37.5	15	62.5	24	18.2		10	62.5	6	37.5	16	16.7	
Progressive disease	0	0	7	100	7	5.3		5	55.6	4	44.4	9	9.4	
Missing data					159									
**CA-125 response**														
Response (decrease)	48	33.3	46	66.7	94	77.7	0.104[b]							
No response (stable/increase)	9	51.1	18	48.9	27	22.3								
Missing data					170									
**Second-line chemotherapy**														
Plat monotherapy	17	68.0	8	32.0	25	17.9	0.086[a]							
Taxanes monotherapy	1	16.7	5	83.3	6	4.3								
TopI inhibitor monotherapy	0	0	4	100	4	2.9								
ACs monotherapy	1	50.0	1	50.0	2	1.4								
Plat + taxanes	32	65.3	17	34.7	49	35.0								
Plat + antimetabolites	10	50.0	10	50.0	20	14.3								
Plat + TopI inhibitor	2	66.7	1	33.3	3	2.1								
Plat + ACs	14	60.9	9	39.1	23	16.4								
Taxane + ACs	1	100	0	0	1	0.7								
Plat + taxanes + anti-VEGF	3	100	0	0	3	2.1								
Plat + taxanes + ACs	2	100	0	0	2	1.4								
Plat + antimetabolites + anti-VEGF	1	50.0	1	50.0	2	1.4								
Missing data					151									
**Second-line chemotherapy class**														
Cp monotherapy	17	68.0	8	32.0	25	17.9	0.368[b]							
Other regiments +/- Cp	67	58.3	48	41.7	115	82.1								
Missing data					151									
**PFS time for first relapse (days)**														
>327	139	67.1	68	32.9	207	71.1	**<0.001[b]**							
≤327	20	23.8	64	76.2	84	28.9								

[a]Mann-Whitney; [b]Chi-Square; [c]Fisher's Exact test. The percentage '%' values represent column percentages.
Abbreviation: ACs, *anthracyclines;* ***CA-125,*** *Cancer antigen 125;* ***Cp,*** *carboplatin;* ***ECOG,*** *Eastern cooperative oncology group;* ***EGFR-TKi,*** *epidermal growth factor receptor tyrosine kinase inhibitor;* ***FIGO;*** *International federation of gynecology and obstetrics;* ***MCOA,*** *Mixed cell ovarian adenocarcinoma;* ***NOS,*** *non-specific;* ***PFS,*** *Progression-free survival;* ***Plat,*** *platinum;* ***RECIST,*** *Response evaluation criteria in solid tumors;* ***STKi,*** *serine-threonine kinase inhibitors;* ***TopI,*** *topoisomerase I;* ***TopII,*** *topoisomerase II;* ***VEGF,*** *Vascular endothelial growth factor.* ***Notes:*** *+/- Cp' means with or without carboplatin since some patients did not receive Carboplatin as their primary therapy, and 'other' means other regimens of chemotherapy beside carboplatin. Taxane (e.g., paclitaxel and docetaxel), TopII inhibitor (e.g., etoposide), ACs (e.g., liposomal doxorubicin and epirubicin), Alkylating agents (e.g., cyclophosphamide), STKi (e.g., enzastaurin), EGFR-TKi (e.g., erlotinib and sorafenib), anti-VEGF (e.g., bevacizumab), TopI inhibitor (e.g., topotecan), Antimetabolites (e.g., gemcitabine)*

Table S5. Multivariate logistic regression analysis of factors associated with Class-1 biomarker (negative epigenetic changes and poor outcome) using available data in cohorts (combined total n = 180 patients).

**Clinicopathological features**	**uOR (95%CI)**	**p-value**	**aOR (95%CI)**	**p-value**
**Age at relapse (n=291, years)**				
Elderly (≥75) vs younger (<75) [*Ref*]	1.41 (0.69-2.89)	0.345	1.14 (0.29-4.54)	0.851
**FIGO stage (n=291)**				
Advanced (III-IV) vs early (I-II) [*Ref*]	2.31 (1.03-5.19)	0.038	1.43 (0.49-4.20)	0.512
**Histological group (n=291)**				
Non-serous carcinoma vs serous carcinoma [*Ref*]	1.54 (0.93-2.56)	0.095	1.10 (0.54-2.22)	0.799
**First-line chemotherapy class (n=229)**				

Cp with combination vs Cp monotherapy [*Ref*]	1.69 (0.87-3.26)	0.118	∞	N/A
**Residual tumour after surgery (n=228)**				
Any residual tumour vs no residual tumour [*Ref*]	1.86 (1.08-3.19)	0.024	0.80 (0.40-1.62)	0.540
**PFS time, first-relapse period (n=291, days)**				
≤327 vs. >327 [*Ref*]	6.54 (3.66-11.68)	<0.001	6.33 (3.12-12.84)	<0.001

The symbol '∞' denotes an infinite value resulting from the presence of a zero value in a 2x2 column when variables are adjusted together. ***Abbreviation: 95% CI,*** *95% Confidence interval;* ***aOR,*** *Adjusted odds ratio;* ***FIGO,*** *International federation of gynecology and obstetrics;* ***PFS,*** *Progression-free survival;* ***uOR,*** *Unadjusted odd ratio.*

Table S6. The distribution of second-line chemotherapy regimens based on their grouping by chemotherapy class and biomarker status.

Chemotherapy regiments	Chemotherapy class and biomarker status								Total	
	Class 2, Cp Only		Class 2, Other +/- Cp		Class 1, Cp Only		Class 1, Other +/- Cp			
	n	%	n	%	n	%	n	%	n	%
Carboplatin	13	100%	0	0	6	100	0	0	19	18.3
Paclitaxel	0	0	1	2.0	0	0	5	14.3	6	5.8
Liposomal doxorubicin	0	0	1	2.0	0	0	1	2.9	2	1.9
Carboplatin + paclitaxel	0	0	29	58.0	0	0	15	42.9	44	42.3
Carboplatin + gemcitabine	0	0	2	4.0	0	0	5	14.3	7	6.7
Carboplatin + liposomal doxorubicin	0	0	10	20.0	0	0	8	22.9	18	17.3
Paclitaxel + liposomal doxorubicin	0	0	1	2.0	0	0	0	0	1	1.0
Carboplatin + paclitaxel + cediranib	0	0	3	6.0	0	0	0	0	3	2.9
Carboplatin + paclitaxel + epirubicin	0	0	2	4.0	0	0	0	0	2	1.9
Carboplatin + gemcitabine + bevacizumab	0	0	1	2.0	0	0	1	2.9	2	1.9

Notes: +/- Cp' means with or without carboplatin since some patients did not receive Carboplatin as their primary therapy, and 'other' means other regimens of chemotherapy beside carboplatin.

Table S7. Multivariate Cox regression analysis associating overall survival with biomarker class, adjusted by clinical covariates in the **Hammersmith Hospital cohort** (n = 100; only complete data variables).

Clinicopathological features	uHR (95%CI)	p-value	aHR (95%CI)	p-value
**Biomarker status during chemotherapy**				
Class 1 vs class 2 [*Ref*]	1.24 (0.73-2.12)	0.428	1.26 (0.74-2.15)	0.402
**Age at diagnosis (years)**				
Elderly (≥75) vs younger (<75) [*Ref*]	1.97 (0.93-4.18)	0.076	1.97 (0.93-4.18)	0.076
**FIGO stage**				
Advanced (III-IV) vs early (I-II) [*Ref*]	1.14 (0.56-2.32)	0.724	1.28 (0.61-2.72)	0.515
**Histological group**				
Non-serous carcinoma vs serous carcinoma [*Ref*]	1.52 (0.83-2.80)	0.174	1.57 (0.85-2.89)	0.148

Abbreviation: 95%CI*, 95% confidence interval****, aHR****, adjusted hazard ratio,* ***uHR,*** *unadjusted hazard ratio.*

Table S8. Multivariate Cox regression analysis associating overall survival after relapse with biomarker class, adjusted by clinical covariates in **five cohort datasets** (n=291; only complete data variables).

Clinicopathological features	uHR (95%CI)	p-value	aHR (95%CI)	p-value
**Biomarker status at relapse**				

Class 1 vs class 2 [*Ref*]	2.28 (1.73-3.00)	**<0.001**	1.82 (1.35-2.46)	**<0.001**
**Age at relapse (years)**				
Elder (≥75) vs younger (<75) [*Ref*]	1.29 (0.83-2.02)	0.257	1.18 (0.75-1.86)	0.463
**FIGO stage**				
Advanced (III-IV) vs early (I-II) [*Ref*]	1.73 (1.06-2.81)	**0.028**	1.87 (1.13-3.08)	**0.014**
**Histological group**				
Non-serous carcinoma vs serous carcinoma [*Ref*]	1.97 (1.48-2.63)	**<0.001**	1.82 (1.33-2.50)	**<0.001**
**PFS time, first-relapse period (days)**				
≤327 vs. >327 [*Ref*]	2.80 (2.07-3.78)	**<0.001**	1.87 (1.33-2.64)	**<0.001**

***Abbreviation: 95%CI**, 95% confidence interval**, aHR**, adjusted hazard ratio, **FIGO,** International federation of gynecology and obstetrics; **PFS,** Progression-free survival; **uHR,** unadjusted hazard ratio.*

Table S9. Survival rate and median follow-up differences of patients between two classes of PLAT-M8 in the six included cohorts.

**Survival outcomes**	**Survival rate[a] and median of follow up (95%CI) across different class of biomarker**							
	**Relapse (ScoTROC 1D & -1V, OCTIPS, BriTROC 1, OV04)**				**Non-relapse (HH cohort)[b]**			
	**Class 2**	**Class 1**	**Overall cases**	**p-value**	**Class 2**	**Class 1**	**Overall cases**	**p-value**
	N = 159 (54.6%)	N = 132 (45.4%)			N = 67 (67.0%)	N = 33 (33.0%)		
2-year OS rate[c], n = 391	45%	15%	32%	**<0.001[e]**	44%	33%	40%	0.311[e]
Median follow up from initial diagnosis (months), n = 391[d]	77.46 (51.25-103.67)	91.13 (69.01-113.26)	80.71 (62.23-99.19)	0.208[e]	60.53 (46.27-74.78)	63.29 (38.09-88.49)	63.29 (52.95-73.62)	0.667[e]
Median follow up from relapse timepoint (months), n = 291[d]	42.08 (34.23-49.94)	64.37 (61.86-66.89)	48.1 (29.07-67.12)	0.560[e]	N/A	N/A	N/A	N/A

[a]The survival rate was calculated based on life tables, following the cumulative proportion surviving at the end of the interval.
[b]Selected only the first sample from each patient (if there is more than 1 sample given) to avoid duplications in calculating endpoints for the 100 patients as individuals;
[c]OS in five cohorts measures the time from the first relapse to death or loss to follow-up (LTFU). For survivors, OS represents the time to their last follow-up (censored time). In the Hammersmith Hospital study (non-relapse cohort), OS specifically tracks the time from the last registered chemotherapy to death or LTFU;
[d]Median follow-up was calculated using reverse Kaplan Meier;
[e]Log-rank test

***Abbreviations: BriTROC 1**, British translational research ovarian cancer collaborative 1; **HH,** Hammersmith Hospital; **OCTIPS,** Ovarian cancer therapy innovative models prolong survival; **OS,** Overall survival after relapse; **OV04,** Ovarian cancer clinical trial study 4th edition; **PFS,** progression-free survival; **ScoTROC 1,** Scottish randomised trial in ovarian cancer (D, discovery and V, validation).*

Table S10. Median progression-free time and overall survival time, with statistically significant differences in survival probabilities over time for relapsed ovarian cancer across various variables.

**Clinicopathological features**	**Median survival (95%CI), in months**			
	**PFS**	**p-value**	**OS after relapse**	**p-value**
**Biomarker status (at relapse, n = 291) in 5 cohorts**				
Class 2	22.59 (19.50-25.67)	**<0.001**	30.44 (22.71-38.17)	**<0.001**
Class 1	11.77 (11.00-12.54)		11.64 (9.58-13.69)	
**Biomarker status (during chemo, n = 100) in HH cohort**				
Class 2	23.37 (15.82-30.93)	0.127	28.96 (17.60-40.33)	0.428

Class 1	17.75 (12.45-23.06)		23.01 (11.04-34.99)	
**Age at relapse category (n = 291)**				
Younger (≤75 years)	16.54 (13.86-19.21)	0.287	20.55 (15.47-25.63)	0.256
Elder (>75 years)	18.58 (13.55-23.60)		16.57 (8.90-24.24)	
**FIGO stage degree (n = 291)**				
Early (I-II)	27.26 (0-55.18)	**0.004**	46.06 (28.82-63.30)	**0.026**
Advance (III-IV)	15.81 (13.67-17.96)		17.79 (14.17-21.40)	
**Histological group (n = 291)**				
Serous carcinoma	20.71 (17.23-24.20)	**<0.001**	25.31 (20.28-30.35)	**<0.001**
Non-serous carcinoma	11.21 (10.20-12.22)		10.88 (8.33-13.43)	
**First-line chemo (n = 229)**				
Cp monotherapy	27.81 (0-57.33)	**0.003**	21.17 (12.07-30.27)	0.354
Cp +/- other regiments	24.79 (21.59-27.99)		16.11 (10.50-21.72)	
**Platinum sensitivity (n = 47)**				
Sensitive	28.54 (22.62-34.45)	**<0.001**	25.74 (16.50-34.98)	**<0.001**
Resistant	9.73 (8.89-10.58)		11.14 (2.12-20.17)	
**Surgical type (n = 57)**				
Interval debulking	22.03 (14.69-29.36)	0.817	21.90 (16.93-26.86)	0.728
Primary debulking	20.09 (0.96-39.22)		17.49 (0-44.62)	
**Residual disease (n = 228)**				
No residual disease	22.03 (17.74-26.31)	**<0.001**	32.61 (24.61-40.62)	**<0.001**
Any residual disease	12.00 (11.21-12.79)		13.51 (11.54-15.49)	
**CA-125 response (n = 121)**				
Response (decrease)	11.93 (11.23-12.64)	**0.004**	12.62 (9.75-15.49)	0.697
No response (stable/increase)	7.60 (1.41-13.678)		8.25 (3.51-12.99)	
**Second-line chemo (n = 104)**				
Cp monotherapy	26.83 (0-82.35)	0.064	38.00 (16.31-59.70)	0.238
Other regiments +/- Cp	22.13 (16.76-27.49)		18.48 (14.14-22.82)	
**Biomarker, second-line chemo (n = 104)**				
Class 2, Cp only	99.55 (0-205.19)	**<0.001**	38.07 (37.91-38.23)	**<0.001**
Class 2, others +/- Cp	24.85 (20.81-28.90)		25.74 (20.44-31.04)	
Class 1, Cp only	14.79 (8.84-20.75)		11.14 (0.96-12.70)	
Class 1, others +/- Cp	13.38 (11.35-15.41)		16.57 (13.91-19.22)	
**Biomarker, second-line chemo – Cp only (n = 19)**				
Class 2, Cp only	99.55 (0-205.19)	**0.001**	38.07 (37.91-38.23)	**<0.001**
Class 1, Cp only	14.79 (8.84-20.75)		11.14 (0.96-12.70)	

Abbreviation: CA-125, *Cancer antigen 125;* ***Cp,*** *carboplatin;* ***FIGO;*** *International federation of gynaecology and obstetrics;* ***PFS,*** *Progression-free survival.*

Supplementary figures

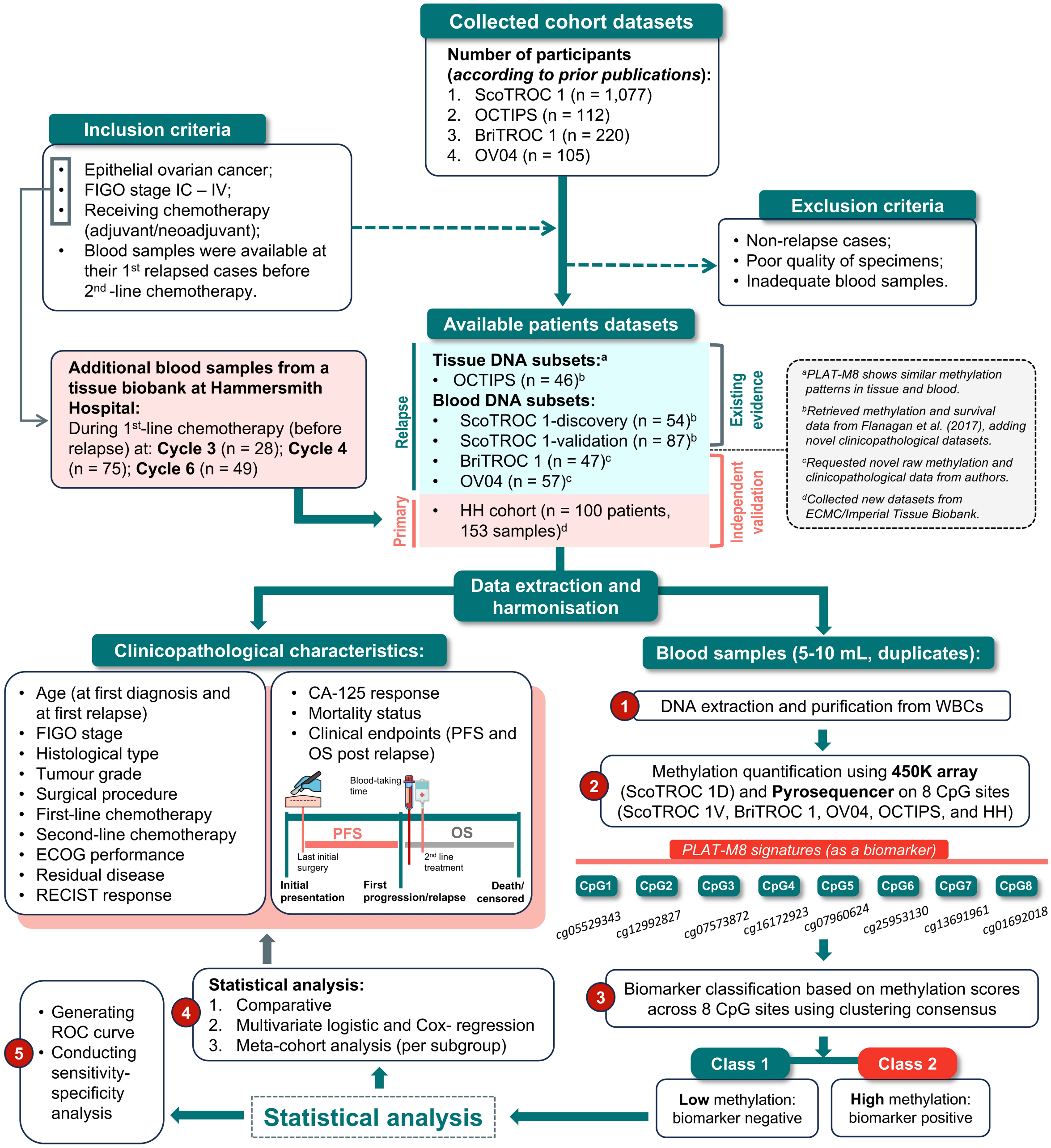

Figure S1. Flow diagram of the PLAT-M8 clinical validation study. The study population includes relapsed ovarian cancer patients from five datasets (with ScoTROC-1 divided into two cohorts) and non-relapsed samples from Hammersmith Hospital. Inclusion and exclusion criteria were applied to select cases, extract clinical data, and analyse stored blood samples for DNA methylation using targeted sequencing with the PLAT-M8 biomarker. Biomarker status was categorised into two classes based on methylation levels: Class 1 (low or absent methylation) and Class 2 (high methylation). Correlations with clinicopathological characteristics were analysed. The number of patients (n) at each selection stage and reasons for exclusion are detailed. *Abbreviations: BriTROC-1, British translational research ovarian cancer collaborative 1; ECOG: Eastern cooperative oncology group performance status; FIGO, International Federation of Gynecology and Obstetrics; HH, Hammersmith Hospital; OCTIPS, Ovarian cancer therapy innovative models prolong survival; OS, Overall survival after relapse; OV04, Ovarian cancer clinical trial study 4th edition; PFS, progression-free survival; PFI, platinum-free interval; PFS, Progression-free survival; RECIST, Response evaluation criteria in solid tumours; ScoTROC-1, Scottish randomised trial in ovarian cancer (D, discovery and V, validation).*

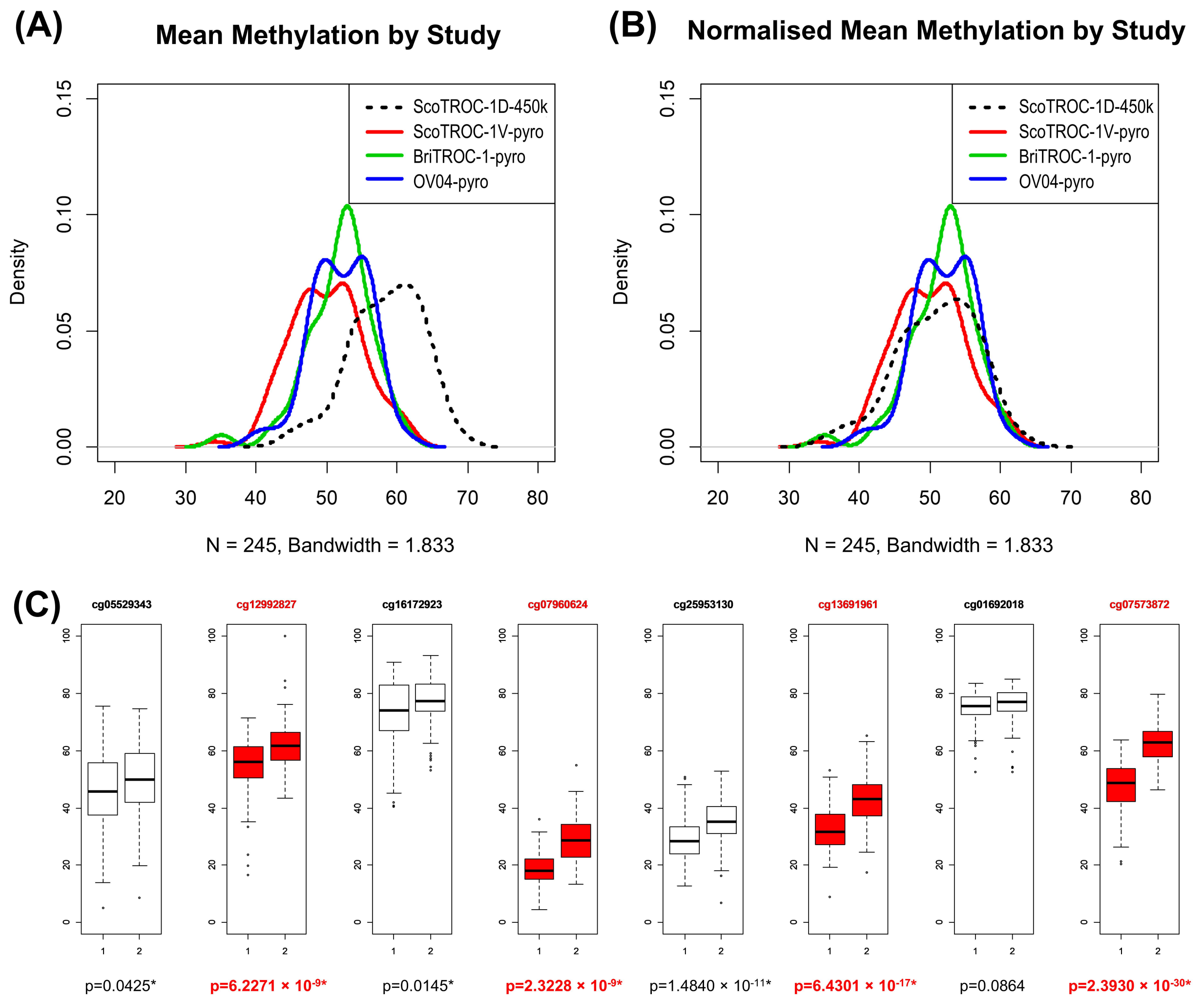

Figure S2. Quality control and methylation analysis of blood samples from relapsed ovarian cancer patients across four cohorts. (Note: OCTIPS study excluded due to methylation data originating from tissue biopsy, not blood.) Bandwidth: 1.833. DNA sample numbers are indicated for each cohort (ScoTROC 1-450K = 54, ScoTROC 1-pyro = 87, BriTROC 1-pyro = 47, OV04 = 57). **(A)** ScoTROC 1 shows distinct mean methylation (%) distribution due to the different methylation analysis techniques (450K methylation array was used in their discovery process and pyrosequencing was used in the validation). **(B)** The distribution of mean DNA methylation percentages following normalisation is depicted in the figure, with a black dashed line indicating the adjusted average through reference normalisation. Prior to normalisation, ScoTROC 1D-450K exhibited a median of 58.88% (IQR: 54.53-62.44%) and a mean of 58.46% (Min. 44.64%, Max. 68.96%). After normalisation, ScoTROC 1D-450K showed a median of 51.47% (IQR: 46.64-55.58%) and a mean of 51.05% (Min. 36.00%, Max. 63.01%). Other cohorts post-normalisation included ScoTROC 1V-pyro (Median 49.85%, IaQR: 46.48-50.01%, Mean 53.15%, Min. 34.18%, Max. 61.20%), BriTROC 1-pyro (Median 52.56%, IQR: 48.93-54.19%, Mean 51.81%, Min. 35.07%, Max. 60.99%), and OV04-pyro (Median 51.98%, IQR: 48.61-55.10%, Mean 52.02%, Min. 39.81%, Max. 61.55%). Most probes showed methylation levels between 30% and 70% at a density level of 0.05 to 0.1, with a prominent peak mean around 52% DNA methylation. BriTROC 1 and ScoTROC 1-450K had a single peak, whereas ScoTROC 1-pyro and OV04-pyro displayed dual peaks. The normalisation process involved centring and scaling. **(C)** All CpG sites were examined across 245 DNA samples from four cohorts. Highlighted in red are the four CpG sites selected using Elastic Net: cg12992827, cg21625271, cg07960624, and cg13691961. Hypermethylation was observed in 7 out of 8 CpG sites (Wilcoxon Test, $p < 0.05$, indicated by an asterisk). Each boxplot shows % methylation (Y-axis) for the n samples from the Infinium HumanMethylation450k BeadChip and pyrosequencer after normalisation for class 1 and class 2 samples (x-axis).

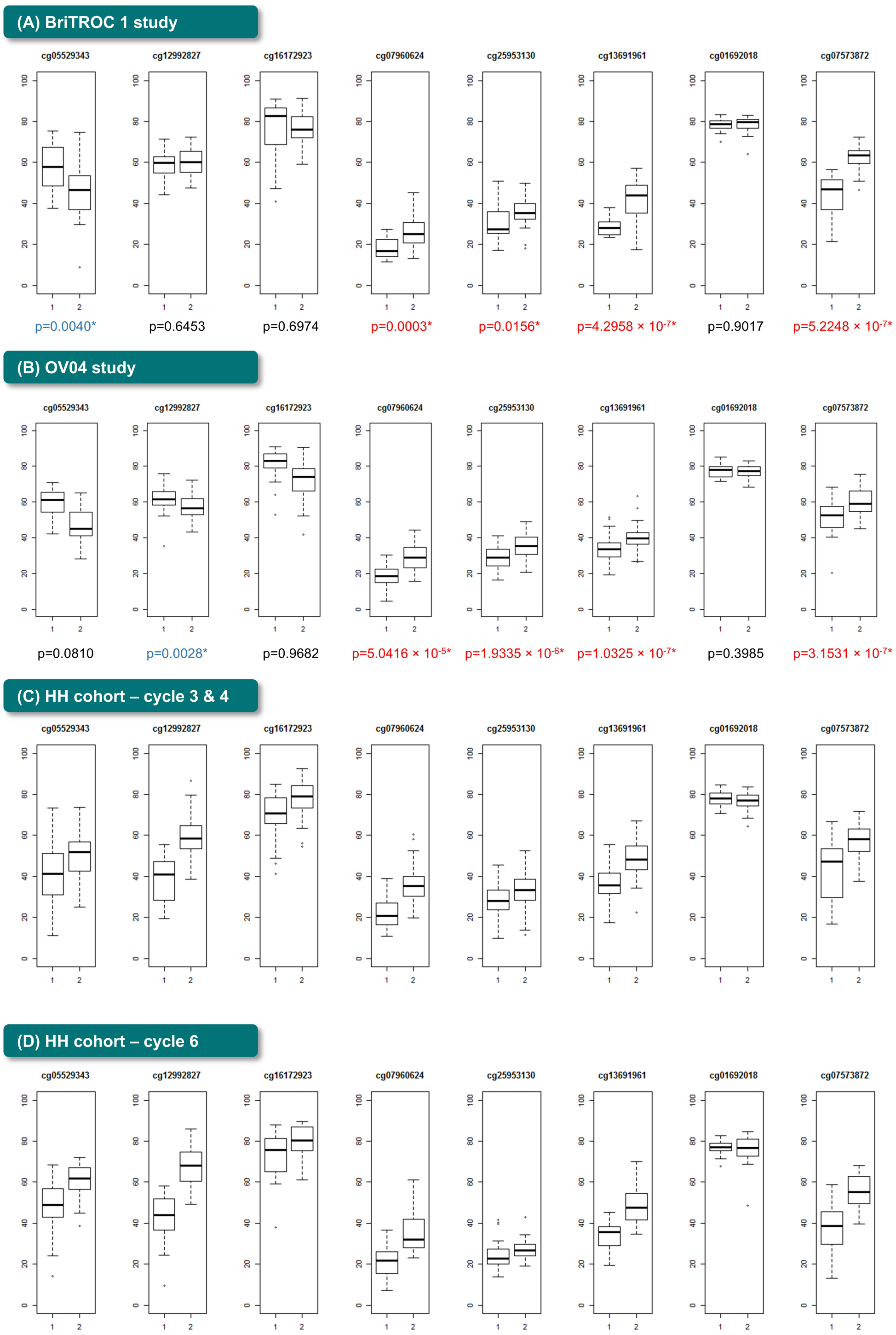

Figure S3. Methylation levels (%) in blood DNA from relapsed and non-relapsed ovarian cancer patients, comparing Class 1 and Class 2 of the PLAT-M8 biomarker using a pyrosequencer. **(A)** BriTROC 1 study (n = 16, Class 1; n = 31, Class 2) shows significant hypermethylation in Class 2 at 4/8 CpG sites ($p < 0.05$, indicated by an asterisk and red), and hypomethylation at 1/8 CpG sites ($p < 0.05$, indicated by an asterisk and blue). **(B)** OV04 study (n = 25, Class 1; n = 32, Class 2) reveals similar patterns with 4/8 CpG sites hypermethylated ($p < 0.05$, indicated by an asterisk and red) and 1/8 CpG sites hypomethylated ($p < 0.05$, indicated by an asterisk and blue). **(C)** Hammersmith Hospital cycle 3&4 study (n = 52, Class 1; n = 51, Class 2) does not show significant differences in methylation **(D)** Hammersmith Hospital cycle 6 study (n = 24, Class 1; n = 26, Class 2) does not show significant differences in methylation.

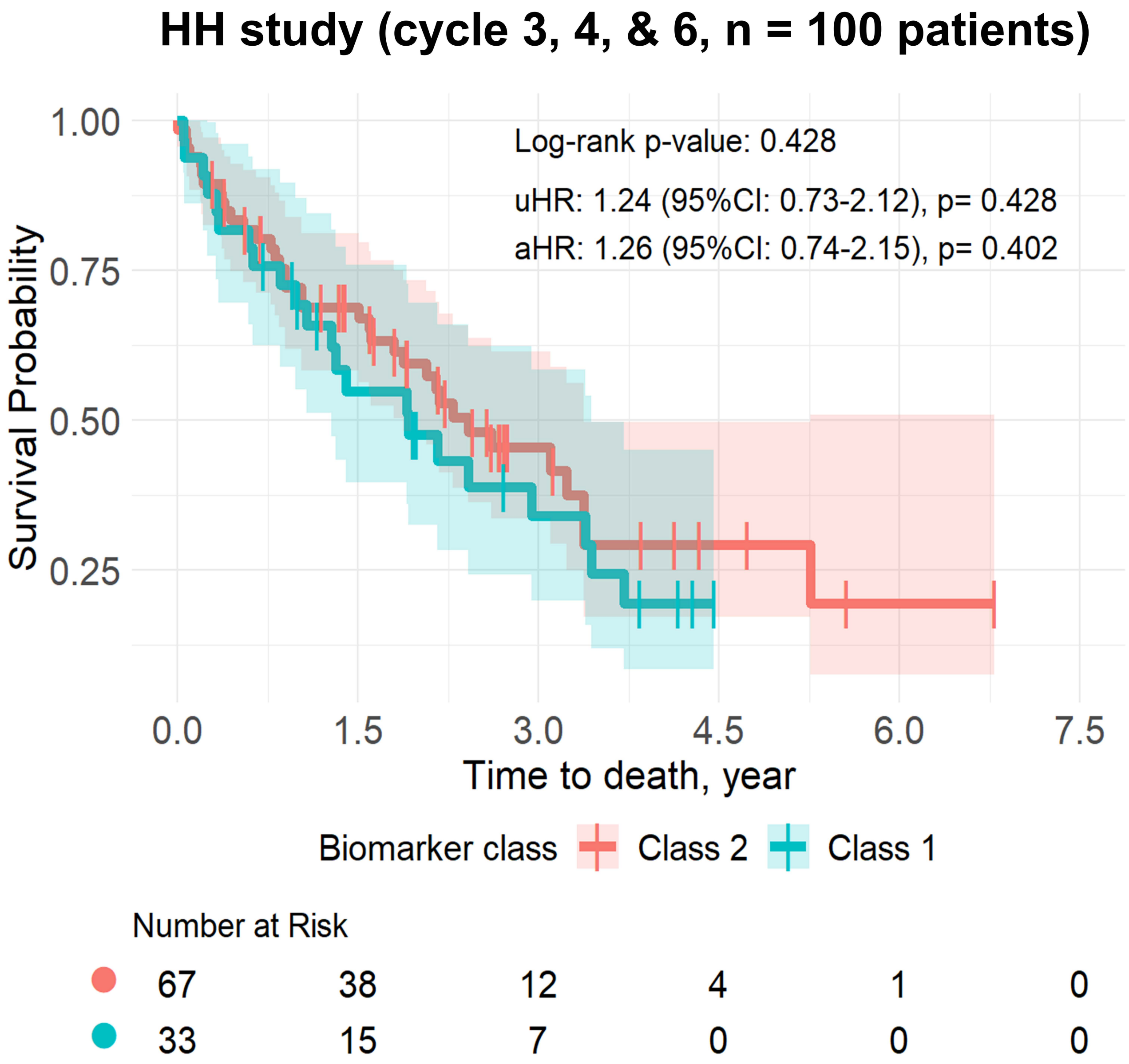

Figure S4. Methylation markers are not prognostic for ovarian cancer survival during first-line chemotherapy (analysis focuses on 100 patients, excluding cycle-specific data). Blood samples were collected from the Hammersmith Hospital cohort during the first-line chemotherapy (Carboplatin + Paclitaxel) treatment course. For this analysis, we selected only the initial sample from each patient to avoid duplications in calculating endpoints for the 100 patients. This analysis differs from the prior figure, which included 153 samples. Analysing 100 patients, overall survival (OS) after relapse did not show significant differences between Class 1 (n = 33) and Class 2 (n = 67) of PLAT-M8. However, there is a tendency that Class 1 might have worse survival. All adjustments were made for the covariates of age at diagnosis, cancer stage, histology, and progression-free survival (PFS).

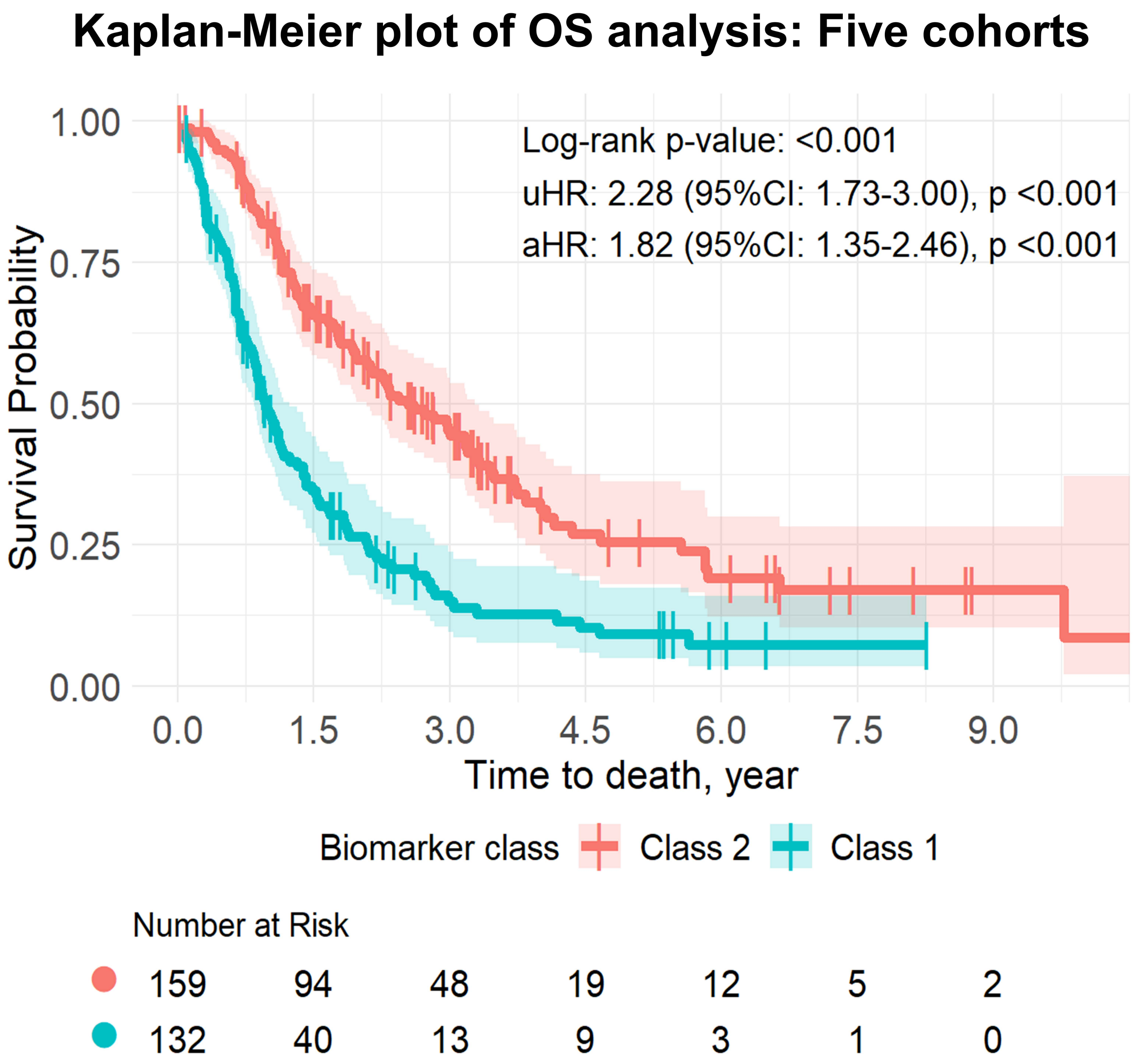

Figure S5. PLAT-M8 validation in five cohorts with harmonised clinicopathological characteristics and normalised methylation data (ScoTROC-1D, ScoTROC-1V, and OCTIPS from Flanagan et al. 2017 along with the addition of novel independent validation cohorts: BriTROC-1 and OV04 studies). In 291 patients across the five cohorts, Kaplan-Meier curves for relapse methylation Class 1 (blue, n = 138) versus Class 2 (red, n = 153) revealed a multivariable adjusted Cox regression for overall survival (OS) with an adjusted hazard ratio (aHR) of 1.82 (95% CI: 1.35-2.46, p < 0.001), log-rank p < 0.001. All adjustments were made for the covariates of age at relapse, cancer stage, histology, and progression-free survival (PFS).

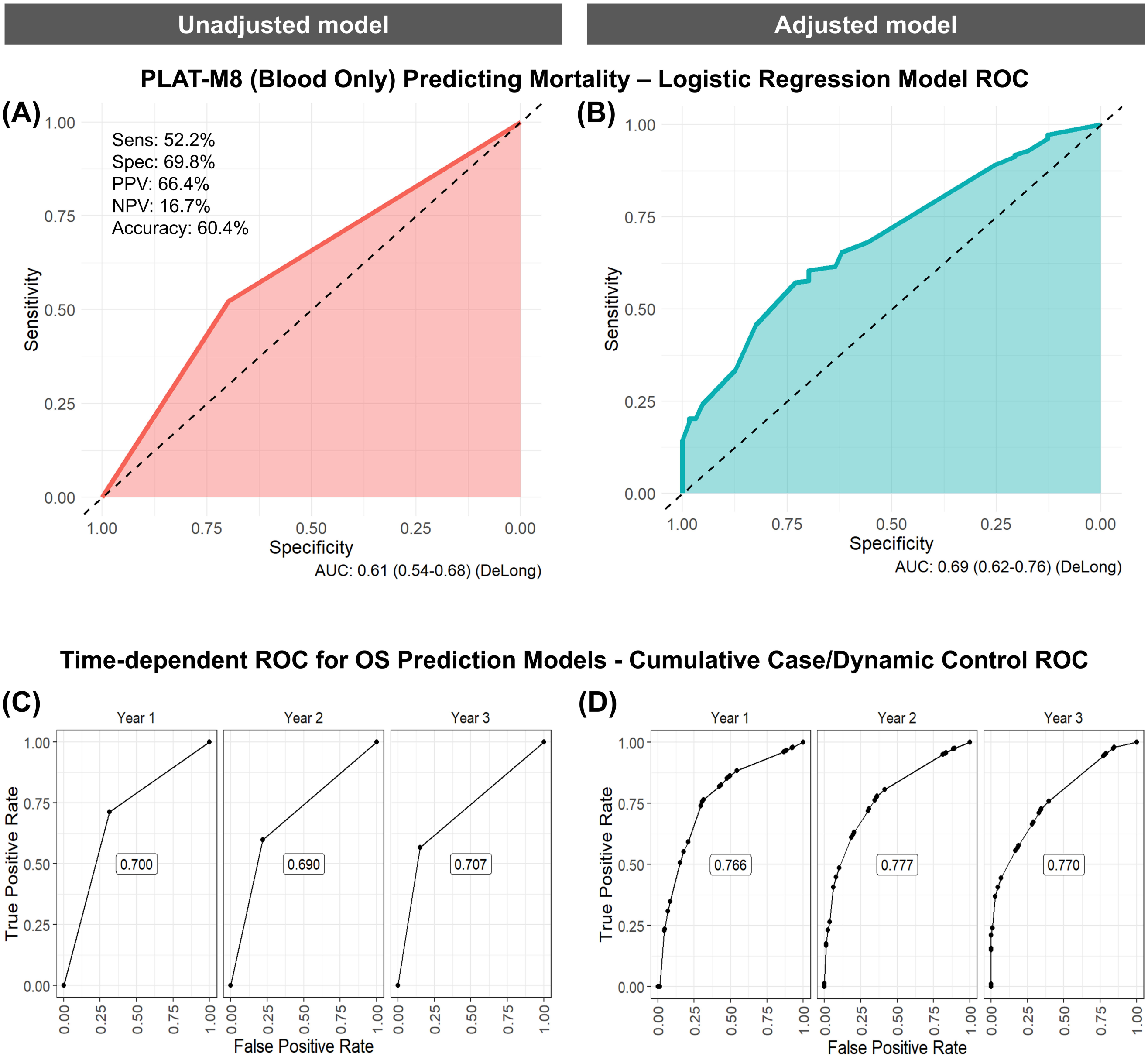

Figure S6. Assessing the prognostic performance of PLAT-M8 class 1 vs. class 2 (reference) in blood DNA samples to predict mortality and time-dependent survival among relapsed cases (n = 245). **(A)** Using a univariate logistic regression model to predict mortality, PLAT-M8 alone has a sensitivity of 52.2%, specificity of 69.8%, a positive predictive value (PPV) of 66.4%, a negative predictive value (NPV) of 16.7%, and an accuracy of 60.4% with an AUC of 0.61. **(B)** Using a multivariate logistic regression model involving age at relapse, FIGO stage, histological type of tumour, and PFS time, PLAT-M8 may predict mortality with an improved AUC of 0.69. **(C)** Using a univariate Cox-regression model to assess time-dependent overall survival (OS) prediction, the performance of PLAT-M8 in predicting the cumulative incidence (events) risk illustrates its superior discriminative capability over 3 years. It excels particularly in the first year, boasting an AUC of 0.700, and experiences a slight increase to 0.707 by the third year. **(D)** After adjustment, PLAT-M8 maintains a consistent discriminative value, with an AUC of 0.766 persisting from the first year to the third year.

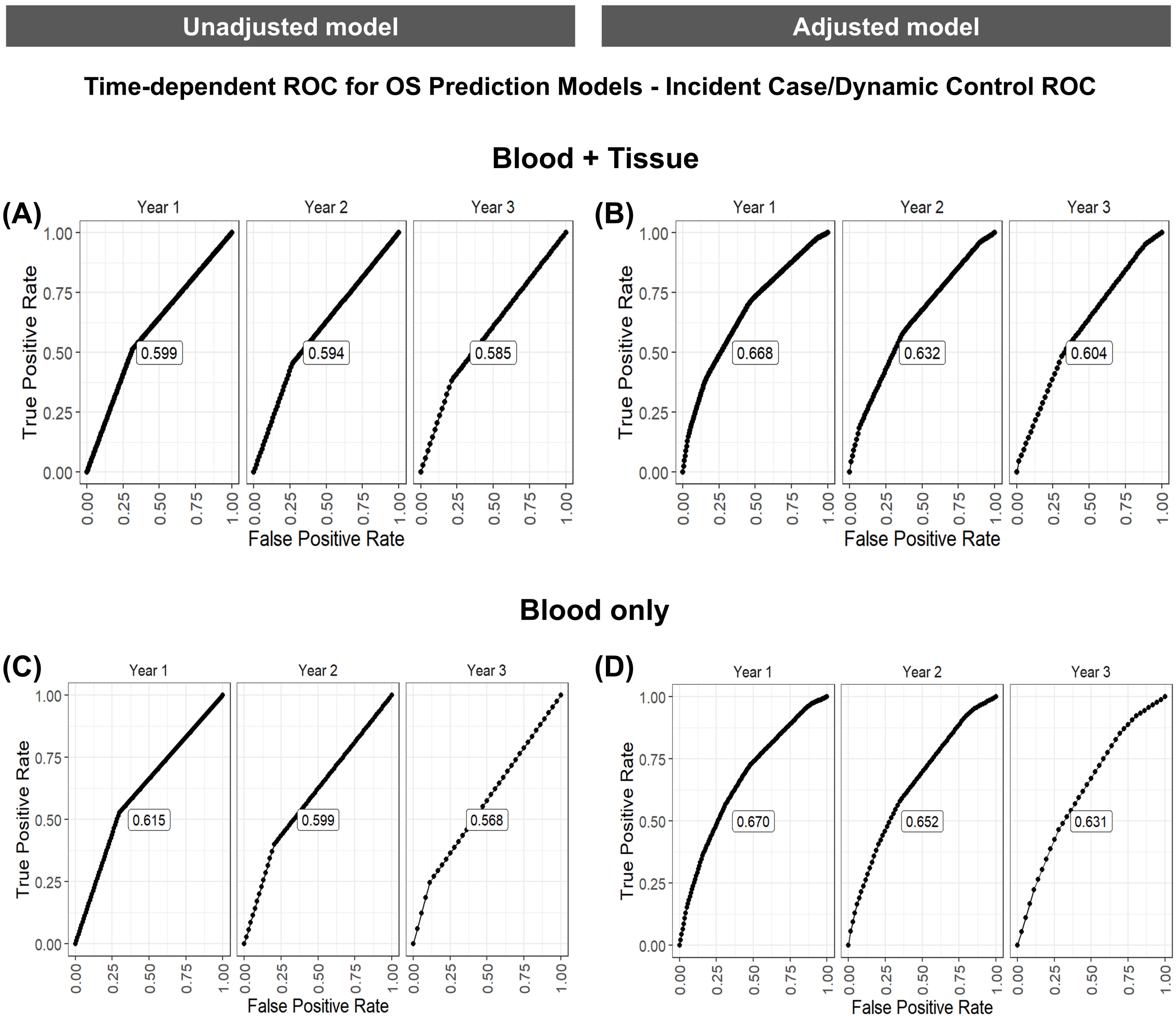

Figure S7. Time-dependent ROC curves for survival prediction models using blood and tissue biopsy DNA samples (n = 291) and blood DNA samples only (n = 245). **(A)** The univariate Cox-regression model for time-dependent overall survival (OS) prediction using PLAT-M8 demonstrates sufficient discriminative value, with an initial AUC of 0.599 in the first year and a slight decrease to 0.585 by the third year for predicting hazards at the 3-year mark. **(B)** After adjustment involving age at relapse, FIGO stage, histological type of tumour, and PFS time, PLAT-M8 improves its discriminative value in the first year with an AUC of 0.668, decreasing over time to 0.604 in the third year. **(C)** The univariate Cox-regression model for time-dependent OS prediction using PLAT-M8 demonstrates sufficient discriminative value, with an initial AUC of 0.615 in the first year and a slight decrease to 0.568 by the third year for predicting hazards at the 3-year mark. **(D)** After adjustment involving age at relapse, FIGO stage, histological type of tumour, and PFS time, PLAT-M8 improves its discriminative value in the first year with an AUC of 0.670, decreasing over time to 0.631 in the third year.

References

1. Flanagan JM, Wilson A, Koo C, Masrour N, Gallon J, Loomis E, Flower K, Wilhelm-Benartzi C, Hergovich A, Cunnea P, Gabra H, Braicu EI, et al. Platinum-Based Chemotherapy Induces Methylation Changes in Blood DNA Associated with Overall Survival in Patients with Ovarian Cancer. Clin Cancer Res 2017;23: 2213-22.
2. Sauer CM, Hall JA, Couturier DL, Bradley T, Piskorz AM, Griffiths J, Sawle A, Eldridge MD, Smith P, Hosking K, Reinius MAV, Morrill Gavarro L, et al. Molecular landscape and functional characterization of centrosome amplification in ovarian cancer. Nat Commun 2023;14: 6505.
3. Vasey PA, Jayson GC, Gordon A, Gabra H, Coleman R, Atkinson R, Parkin D, Paul J, Hay A, Kaye SB, Scottish Gynaecological Cancer Trials G. Phase III randomized trial of docetaxel-carboplatin versus paclitaxel-carboplatin as first-line chemotherapy for ovarian carcinoma. J Natl Cancer Inst 2004;96: 1682-91.
4. Vasey PA. Carboplatin Plus Paclitaxel or Docetaxel in Treating Patients With Ovarian Epithelial Cancer, vol. 2023 University of Glasgow: National Cancer Institute (NCI), 1998.
5. Barrett SV, Paul J, Hay A, Vasey PA, Kaye SB, Glasspool RM, Scottish Gynaecological Cancer Trials G. Does body mass index affect progression-free or overall survival in patients with ovarian cancer? Results from SCOTROC I trial. Ann Oncol 2008;19: 898-902.
6. Crawford SC, Vasey PA, Paul J, Hay A, Davis JA, Kaye SB. Does aggressive surgery only benefit patients with less advanced ovarian cancer? Results from an international comparison within the SCOTROC-1 Trial. J Clin Oncol 2005;23: 8802-11.
7. He YJ, Winham SJ, Hoskins JM, Glass S, Paul J, Brown R, Motsinger-Reif A, McLeod HL. Carboplatin/taxane-induced gastrointestinal toxicity: a pharmacogenomics study on the SCOTROC1 trial. Pharmacogenomics J 2016;16: 243-8.
8. Lambrechts S, Smeets D, Moisse M, Braicu EI, Vanderstichele A, Zhao H, Van Nieuwenhuysen E, Berns E, Sehouli J, Zeillinger R, Darb-Esfahani S, Cacsire Castillo-Tong D, et al. Genetic heterogeneity after first-line chemotherapy in high-grade serous ovarian cancer. Eur J Cancer 2016;53: 51-64.
9. Stanske M, Wienert S, Castillo-Tong DC, Kreuzinger C, Vergote I, Lambrechts S, Gabra H, Gourley C, Ganapathi RN, Kolaschinski I, Budczies J, Sehouli J, et al. Dynamics of the Intratumoral Immune Response during Progression of High-Grade Serous Ovarian Cancer. Neoplasia 2018;20: 280-8.
10. Ruscito I, Cacsire Castillo-Tong D, Vergote I, Ignat I, Stanske M, Vanderstichele A, Ganapathi RN, Glajzer J, Kulbe H, Trillsch F, Mustea A, Kreuzinger C, et al. Exploring the clonal evolution of CD133/aldehyde-dehydrogenase-1 (ALDH1)-positive cancer stem-like cells from primary to recurrent high-grade serous ovarian cancer (HGSOC). A study of the Ovarian Cancer Therapy-Innovative Models Prolong Survival (OCTIPS) Consortium. Eur J Cancer 2017;79: 214-25.
11. Bartl T, Karacs J, Kreuzinger C, Pfaffinger S, Kendler J, Ciocsirescu C, Wolf A, Reinthaller A, Meyer E, Brandstetter M, Postl M, Langthaler E, et al. Tumor Growth Rate Estimates Are Independently Predictive of Therapy Response and Survival in Recurrent High-Grade Serous Ovarian Cancer Patients. Cancers (Basel) 2021;13.
12. Ruscito I, Cacsire Castillo-Tong D, Vergote I, Ignat I, Stanske M, Vanderstichele A, Glajzer J, Kulbe H, Trillsch F, Mustea A, Kreuzinger C, Benedetti Panici P, et al. Characterisation of tumour microvessel density during

progression of high-grade serous ovarian cancer: clinico-pathological impact (an OCTIPS Consortium study). Br J Cancer 2018;119: 330-8.

13. Glajzer J, Castillo-Tong DC, Richter R, Vergote I, Kulbe H, Vanderstichele A, Ruscito I, Trillsch F, Mustea A, Kreuzinger C, Gourley C, Gabra H, et al. Impact of BRCA Mutation Status on Tumor Dissemination Pattern, Surgical Outcome and Patient Survival in Primary and Recurrent High-Grade Serous Ovarian Cancer: A Multicenter Retrospective Study by the Ovarian Cancer Therapy-Innovative Models Prolong Survival (OCTIPS) Consortium. Ann Surg Oncol 2023;30: 35-45.
14. Goranova T, Ennis D, Piskorz AM, Macintyre G, Lewsley LA, Stobo J, Wilson C, Kay D, Glasspool RM, Lockley M, Brockbank E, Montes A, et al. Safety and utility of image-guided research biopsies in relapsed high-grade serous ovarian carcinoma-experience of the BriTROC consortium. Br J Cancer 2017;116: 1294-301.
15. McNeish IA, Brenton JD. A study to help understand why ovarian cancer can come back or continue to grow after treatment (BriTROC 1), vol. 2023 University of Cambridge: Cancer Research UK, 2012.
16. Schwarz RF, Ng CK, Cooke SL, Newman S, Temple J, Piskorz AM, Gale D, Sayal K, Murtaza M, Baldwin PJ, Rosenfeld N, Earl HM, et al. Spatial and temporal heterogeneity in high-grade serous ovarian cancer: a phylogenetic analysis. PLoS Med 2015;12: e1001789.
17. Schwarz RF, Trinh A, Sipos B, Brenton JD, Goldman N, Markowetz F. Phylogenetic quantification of intra-tumour heterogeneity. PLoS Comput Biol 2014;10: e1003535.
18. Cheng Z, Mirza H, Ennis DP, Smith P, Morrill Gavarro L, Sokota C, Giannone G, Goranova T, Bradley T, Piskorz A, Lockley M, Bri T-I, et al. The Genomic Landscape of Early-Stage Ovarian High-Grade Serous Carcinoma. Clin Cancer Res 2022;28: 2911-22.
19. Ng CKY, Brenton JD. CTCR-OV03/CTCR-OV04 ovarian cancer: Genome variation profiling by SNP array, vol. 2023 University of Cambridge: NCBI Gene Expression Omnibus, 2012.
20. Brenton JD. A study to look more closely at factors affecting how well chemotherapy works for cancer of the ovary, vol. 2023 University of Cambridge: Cancer Research UK, 2008.
21. Martins FC, Couturier DL, de Santiago I, Sauer CM, Vias M, Angelova M, Sanders D, Piskorz A, Hall J, Hosking K, Amirthanayagam A, Cosulich S, et al. Clonal somatic copy number altered driver events inform drug sensitivity in high-grade serous ovarian cancer. Nat Commun 2022;13: 6360.
22. Liebscher CA, Prinzler J, Sinn BV, Budczies J, Denkert C, Noske A, Sehouli J, Braicu EI, Dietel M, Darb-Esfahani S. Aldehyde dehydrogenase 1/epidermal growth factor receptor coexpression is characteristic of a highly aggressive, poor-prognosis subgroup of high-grade serous ovarian carcinoma. Hum Pathol 2013;44: 1465-71.
23. Eisenhauer EA, Therasse P, Bogaerts J, Schwartz LH, Sargent D, Ford R, Dancey J, Arbuck S, Gwyther S, Mooney M, Rubinstein L, Shankar L, et al. New response evaluation criteria in solid tumours: revised RECIST guideline (version 1.1). Eur J Cancer 2009;45: 228-47.
24. Rustin GJ, Vergote I Fau - Eisenhauer E, Eisenhauer E Fau - Pujade-Lauraine E, Pujade-Lauraine E Fau - Quinn M, Quinn M Fau - Thigpen T, Thigpen T Fau - du Bois A, du Bois A Fau - Kristensen G, Kristensen G Fau - Jakobsen A, Jakobsen A Fau - Sagae S, Sagae S Fau - Greven K, Greven K Fau - Parmar M, Parmar M Fau - Friedlander M, et al. Definitions for response and progression in ovarian cancer clinical trials incorporating RECIST 1.1 and CA 125 agreed by the Gynecological Cancer Intergroup (GCIG). Int J Gynecol Cancer 2011;21: 419-23.

25. Unal I. Defining an Optimal Cut-Point Value in ROC Analysis: An Alternative Approach. Comput Math Methods Med 2017;2017: 3762651.
26. Dion L, Mimoun C, Nyangoh Timoh K, Bendifallah S, Bricou A, Collinet P, Touboul C, Ouldamer L, Azais H, Dabi Y, Akladios C, Canlorbe G, et al. Ovarian Cancer in the Elderly: Time to Move towards a More Logical Approach to Improve Prognosis-A Study from the FRANCOGYN Group. J Clin Med 2020;9.
27. CRUK. Ovarian cancer statistics, vol. 2023 United Kingdom: Cancer Research UK, 2020.
28. Delgado A, Guddati AK. Clinical endpoints in oncology - a primer. Am J Cancer Res 2021;11: 1121-31.
29. Mankoo PK, Shen R, Schultz N, Levine DA, Sander C. Time to recurrence and survival in serous ovarian tumors predicted from integrated genomic profiles. PLoS One 2011;6: e24709.
30. Baert T, Ferrero A, Sehouli J, O'Donnell DM, Gonzalez-Martin A, Joly F, van der Velden J, Blecharz P, Tan DSP, Querleu D, Colombo N, du Bois A, et al. The systemic treatment of recurrent ovarian cancer revisited. Ann Oncol 2021;32: 710-25.
31. Flanagan JM, Wilhelm-Benartzi CS, Metcalf M, Kaye SB, Brown R. Association of somatic DNA methylation variability with progression-free survival and toxicity in ovarian cancer patients. Ann Oncol 2013;24: 2813-8.
32. Shenker NS, Polidoro S, van Veldhoven K, Sacerdote C, Ricceri F, Birrell MA, Belvisi MG, Brown R, Vineis P, Flanagan JM. Epigenome-wide association study in the European Prospective Investigation into Cancer and Nutrition (EPIC-Turin) identifies novel genetic loci associated with smoking. Hum Mol Genet 2013;22: 843-51.
33. Li H, Chiappinelli KB, Guzzetta AA, Easwaran H, Yen RW, Vatapalli R, Topper MJ, Luo J, Connolly RM, Azad NS, Stearns V, Pardoll DM, et al. Immune regulation by low doses of the DNA methyltransferase inhibitor 5-azacitidine in common human epithelial cancers. Oncotarget 2014;5: 587-98.
34. Du P, Zhang X, Huang CC, Jafari N, Kibbe WA, Hou L, Lin SM. Comparison of Beta-value and M-value methods for quantifying methylation levels by microarray analysis. BMC Bioinformatics 2010;11: 587.
35. Chen YA, Lemire M, Choufani S, Butcher DT, Grafodatskaya D, Zanke BW, Gallinger S, Hudson TJ, Weksberg R. Discovery of cross-reactive probes and polymorphic CpGs in the Illumina Infinium HumanMethylation450 microarray. Epigenetics 2013;8: 203-9.
36. Najmi A, Sadasivam B, Ray A. How to choose and interpret a statistical test? An update for budding researchers. J Family Med Prim Care 2021;10: 2763-7.
37. Cioci AC, Cioci AL, Mantero AMA, Parreco JP, Yeh DD, Rattan R. Advanced Statistics: Multiple Logistic Regression, Cox Proportional Hazards, and Propensity Scores. Surg Infect 2021;22: 604-10.
38. Goel MK, Khanna P, Kishore J. Understanding survival analysis: Kaplan-Meier estimate. Int J Ayurveda Res 2010;1: 274-8.
39. Mikolajewicz N, Komarova SV. Meta-Analytic Methodology for Basic Research: A Practical Guide. Front Physiol 2019;10: 203.
40. Colombo N. Optimising the treatment of the partially platinum-sensitive relapsed ovarian cancer patient. EJC Suppl 2014;12: 7-12.
41. Kamarudin AN, Cox T, Kolamunnage-Dona R. Time-dependent ROC curve analysis in medical research: current methods and applications. BMC Med Res Methodol 2017;17: 53.

42. Sterne JAC, White IR, Carlin JB, Spratt M, Royston P, Kenward MG, Wood AM, Carpenter JR. Multiple imputation for missing data in epidemiological and clinical research: potential and pitfalls. BMJ 2009;338: b2393.
43. Chevret S, Seaman S, Resche-Rigon M. Multiple imputation: a mature approach to dealing with missing data. Intensive Care Med 2015;41: 348-50.
44. Parkinson CA, Gale D, Piskorz AM, Biggs H, Hodgkin C, Addley H, Freeman S, Moyle P, Sala E, Sayal K, Hosking K, Gounaris I, et al. Exploratory Analysis of TP53 Mutations in Circulating Tumour DNA as Biomarkers of Treatment Response for Patients with Relapsed High-Grade Serous Ovarian Carcinoma: A Retrospective Study. PLoS Med 2016;13: e1002198.
45. Sauerbrei W, Taube SE, McShane LM, Cavenagh MM, Altman DG. Reporting Recommendations for Tumor Marker Prognostic Studies (REMARK): An Abridged Explanation and Elaboration. J Natl Cancer Inst 2018;110: 803-11.
46. Cuschieri S. The STROBE guidelines. Saudi J Anaesth 2019;13: S31-S4.
